# Dynamic Assembly and Disassembly of the Human DNA Polymerase δ Holoenzyme on the Genome *In Vivo*

**DOI:** 10.1016/j.celrep.2019.12.101

**Published:** 2020-02-04

**Authors:** William C. Drosopoulos, David A. Vierra, Charles A. Kenworthy, Robert A. Coleman, Carl L. Schildkraut

**Affiliations:** 1 Department of Cell Biology, Albert Einstein College of Medicine, 1300 Morris Park Avenue, Bronx, NY 10461 USA; 2 Department of Anatomy and Structural Biology, Albert Einstein College of Medicine, 1300 Morris Park Avenue, Bronx, NY 10461 USA; 3 Lead Contact

## Abstract

Human DNA polymerase delta (Pol δ) forms a holoenzyme complex with the DNA sliding clamp proliferating cell nuclear antigen (PCNA) to perform its essential roles in genome replication. Here, we utilize live-cell single-molecule tracking to monitor Pol δ holoenzyme interaction with the genome in real time. We find holoenzyme assembly and disassembly *in vivo* are highly dynamic and ordered. PCNA generally loads onto the genome before Pol δ. Once assembled, the holoenzyme has a relatively short lifetime on the genome, implying multiple Pol δ binding events may be needed to synthesize an Okazaki fragment. During disassembly, Pol δ dissociation generally precedes PCNA unloading. We also find that Pol δ p125, the catalytic subunit of the holoenzyme, is maintained at a constant cellular level, indicating an active mechanism for control of Pol δ levels *in vivo*. Collectively, our studies reveal that Pol δ holoenzyme assembly and disassembly follow a predominant pathway *in vivo*; however, alternate pathways are observed.

## In Brief

Drosopoulos et al. report human Pol δ holoenzyme assembly and disassembly on the genome *in vivo* are highly dynamic and ordered. They find that assembly and disassembly of the Pol δ holoenzyme complex follow a predominant pathway *in vivo*, with alternate pathways also observed.

## Graphical Abstract

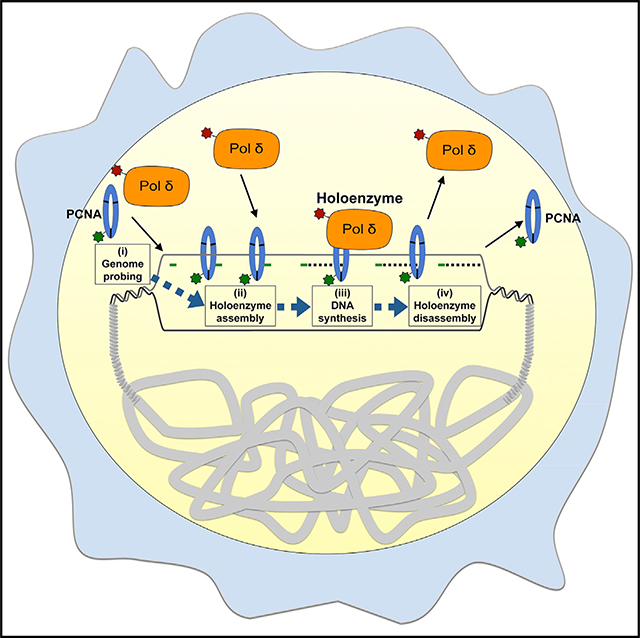

## INTRODUCTION

The eukaryotic genome is replicated primarily by an ensemble of three B-family DNA polymerases, namely, Pol α, Pol δ, and Pol ε (Burgers and Kunkel, 2017). Pol α, together with primase, synthesizes primers that initiate both leading and lagging strand chromosomal replication. Leading strand synthesis is mainly performed by Pol ε, which extends the nascent strand in a continuous uninterrupted manner, whereas the lagging strand is copied in a discontinuous fashion as short (100–250 nt) Okazaki fragments by Pol δ (Burgers and Kunkel, 2017).

**Figure 1.**
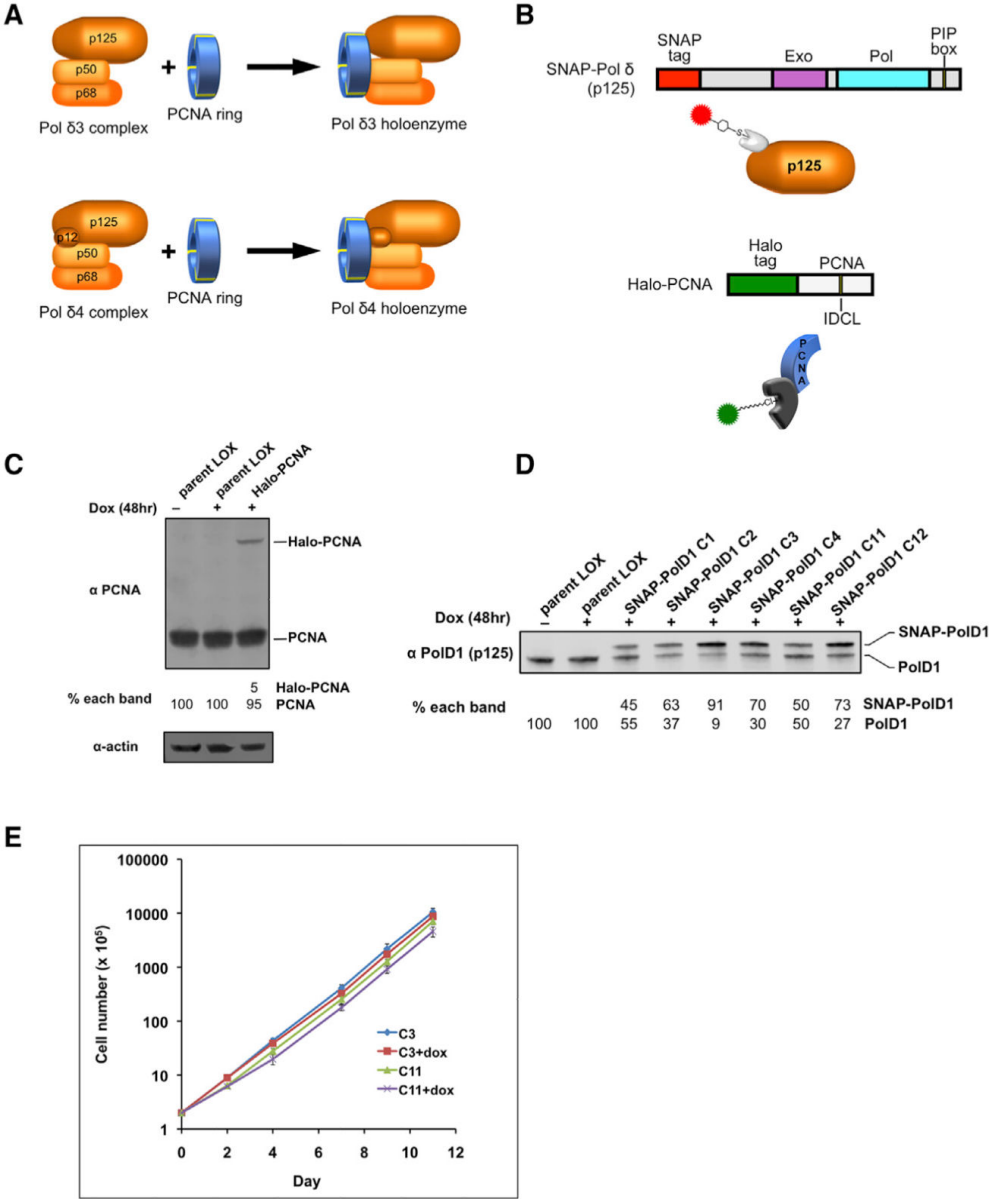
Establishment of a System for Real-Time Imaging of Pol δ Holoenzyme Assembly and Disassembly in Living Human Cells (A) Pol δ holoenzyme complexes formed by Pol δ3 and Pol δ4 assemblies and PCNA trimer ring. (B) Schematic diagram of SNAP-Pol δ p125 and Halo-PCNA fusion proteins (PIP, PCNA interacting protein; IDCL, Interdomain connecting loop). (C) Immunoblot of cell lysates of LOX cell lines stably expressing doxycycline-inducible Halo-PCNA. The percentage of the total PCNA protein represented by Halo-tagged (top band) and untagged endogenous (bottom band) proteins is shown below each lane. (D) Immunoblot of cell lysates of LOX cell lines stably expressing doxycycline-inducible SNAP-Pol δ p125. The percentage of the total Pol δ p125 protein represented by SNAP-tagged (top band) and untagged endogenous (bottom band) proteins is shown below each lane. (E) Growth curves of LOX stable cell lines co-expressing SNAP-Pol δ p125 and Halo-PCNA proteins. Cultures of two clonal lines, C3 (91% of total Pol δ p125 is SNAP-tagged) and C11 (50% of total Pol δ p125 SNAP-tagged), were grown in the absence or presence (+ dox) of 1 μg/ml doxycycline, and cells counted at time points indicated (cell number is relative to initial seeding). Values were obtained from two independent cultures. Error bars indicate SD.

Human Pol δ is a multisubunit enzyme consisting of a catalytic core subunit, p125, encoded by the POLD1 gene (Chung et al., 1991, Chang et al., 1995), which contains the polymerase and exonuclease activities of the enzyme and three accessory subunits (p68, p50, and p12) (Xie et al., 2002). Catalytically active Pol δ complexes can be found in cells as heterotetramers, Pol δ4 (p125/p68/p50/p12), or heterotrimers, Pol δ3 (p125/p68/ p50) (Figure 1A; Lee et al., 2012). Pol δ4 has been shown to have higher polymerase activity than Pol δ3 *in vitro* (Meng et al., 2010; Podust et al., 2002), implying that the δ4 complex could be the major replicative assembly of Pol δ. Pol δ3 is generated *in vivo* by targeted degradation of the p12 subunit in response to UV, alkylating agents, and replication stress (Lee et al., 2012; Zhang et al., 2007), implying it is involved in DNA damage response. However, Pol δ3 is also formed during S phase in unperturbed cells, suggesting that it plays an important role in DNA replication (Chea et al., 2012; Zhang et al., 2013).

As a replicative polymerase, human Pol δ is inherently non-processive, copying only a few bases before releasing from a DNA template. However, the processivity of Pol δ is dramatically increased to the level required for efficient DNA replication by forming a holoenzyme complex with the homotrimeric DNA sliding clamp PCNA (proliferating cell nuclear antigen) (Figure 1A). PCNA tethers Pol δ to the DNA duplex by interactions between the interdomain connecting loop (IDCL) region of PCNA and peptide motifs found on Pol δ subunits known as PIP (PCNA-interacting protein) boxes (Choe and Moldovan, 2017; Slade, 2018). All of the Pol δ subunits contain PIP boxes and interact directly with PCNA (Lee et al., 2017). Because all three subunits of the PCNA homotrimer contain IDCLs, it is possible for multiple Pol δ subunits to interact with the PCNA ring at the same time. Evidence suggests that this is the case, as the apparent K_d_ for PCNA binding *in vitro* increases when either the p12 or the p66 subunit is omitted from the Pol δ complex (Zhou et al., 2012).

The assembly of the human Pol δ holoenzyme is thought to occur in a stepwise manner on DNA *in vitro* (Hedglin et al., 2013). Biochemical studies indicate that the PCNA trimer ring is initially loaded onto the primed DNA template by the multimeric clamp loader replication factor C (RFC) complex. Because RFC and Pol δ interact with the same surface of PCNA, RFC then releases from PCNA, allowing access for Pol δ to load onto DNA and bind PCNA to form the PCNA-Pol δ holoenzyme. Interestingly, *in vitro* studies show that following loading of the PCNA ring, RFC does not dissociate from the DNA template. Rather, RFC remains transiently bound to the DNA near the loaded PCNA (Hedglin et al., 2013). This finding suggests that should Pol δ not complex with PCNA in a timely manner, the DNA-bound RFC can re-engage and unload PCNA from the template. This PCNA recycling mechanism would provide an adequate supply of sliding clamps for ongoing replication of multiple loci in the genome (Hedglin et al., 2013).

More than 30 years of *in vitro* studies have provided valuable insights into mechanisms of human Pol δ holoenzyme enzyme function. However, the dynamics of the Pol δ holoenzyme assembly and disassembly within the living cell on native chromatin is largely unknown. Given the complexity of the nuclear environment, *in vitro* observations may not fully reflect holoenzyme biology in the living system. Recent advances in single-molecule imaging by live-cell single-molecule tracking (SMT) allow for direct observation of individual protein molecules *in vivo* in real time in individual cells (Liu et al., 2015). Therefore, we used live-cell SMT to investigate the dynamics of Pol δ holoenzyme assembly and disassembly on the genome by using fluorescently tagged Pol δ (p125) and PCNA. Our analysis reveals the majority of Pol δ interactions with the genome are short lived (<1.7 s), suggesting transient probing behavior by the polymerase. Longer, stable binding events by Pol δ lasted on average ~13 s, presumably involving productive DNA synthesis. We also detected Pol δ-PCNA colocalizations by SMT that could be disrupted by chemical inhibition of PIP box interactions, indicating colocalization events represent bona fide Pol δ holoenzyme complexes. Analysis of Pol δ-PCNA colocalizations revealed multiple assembly/disassembly pathways of the holoenzyme. We found that PCNA generally assembles on the genome first, followed by Pol δ loading. This *in vivo* assembly order is consistent with Pol δ holoenzyme assembly *in vitro*. We observed holoenzyme disassembly predominantly occurring by Pol δ dissociation from the genome, followed by PCNA unloading. Interestingly, the catalytic activity of Pol δ affected the chromatin binding dynamics of both Pol δ and PCNA. We found overexpression of catalytically dead Pol δ led to increased chromatin residence time of both Pol δ and PCNA during long binding events and increased the duration of colocalization. These findings suggest that the dead polymerase is more stably associated than the active enzyme once loaded onto chromatin and that DNA polymerization may trigger dissociation of the Pol δ complex from chromatin. Collectively, our real-time Pol δ-PCNA colocalization studies indicate that human Pol δ holoenzyme assembly and disassembly on a chromatinized genome in living cells follow an ordered, predominant pathway.

## RESULTS

### Establishment of a System for Real-Time Imaging of Pol δ Holoenzyme Assembly and Disassembly in Living Human Cells

We have developed an approach to track the interactions of Pol δ with nuclear chromatin and PCNA based on the detection of fluorescently labeled Pol δ and PCNA. A tagged version of Pol δ (SNAP-Pol δ) was generated where the 19.5-kDa SNAP protein tag was fused to the N terminus of the catalytic p125 subunit of human Pol δ (Figure 1B). Similarly, the 33-kDa Halo protein tag was fused to the N terminus of the catalytic subunit of human PCNA to generate Halo-PCNA (Figure 1B). These tags can be labeled with highly photostable cell-permeable fluorescent ligand dyes, which form irreversible covalent bonds with the tags. Upon exciting the bound dyes, we can monitor each labeled protein with high temporal and spatial precision, allowing us to track these proteins *in vivo* in real time (Figure 1B).

SNAP-Pol δ and Halo-PCNA proteins were co-expressed under the control of doxycycline-inducible promoters from constructs stably integrated into LOX cells, a human melanoma cell line (Fodstad et al., 1988). Initially we generated a LOX cell line expressing Halo-PCNA only, where the inducible tagged protein represented 5% of total PCNA protein in the cell (Figure 1C). Previous studies have shown that N-terminal EGFP-tagged PCNA was functional and incorporated into active replication foci in live cells and did not disrupt cell growth when expressed at ~11% of total cellular PCNA (Chagin et al., 2016). These cells were then used to generate individual clonal lines co-expressing Halo-PCNA and varied levels of SNAP-tagged p125. Interestingly, the endogenous Pol δ p125 expression levels in the various clones were modulated according to the expression levels of the ectopically expressed SNAP-Pol δ (i.e., endogenous p125 expression was inversely proportional to SNAP-Pol δ expression) (Figure 1D). The net effect was that the total cellular p125 levels (endogenous plus SNAP-tagged p125) remained unchanged. This compensatory effect suggested that the SNAP-p125 was fully functional. In addition, we saw no difference in growth rate upon induction of expression of the tagged proteins, even when the tagged protein constituted 90% of total p125 (Figure 1E), further indicating functional substitution for the endogenous protein.

### *In Vivo* Dynamic Interaction of Pol δ with PCNA Is PIP Box Mediated

**Figure 2.**
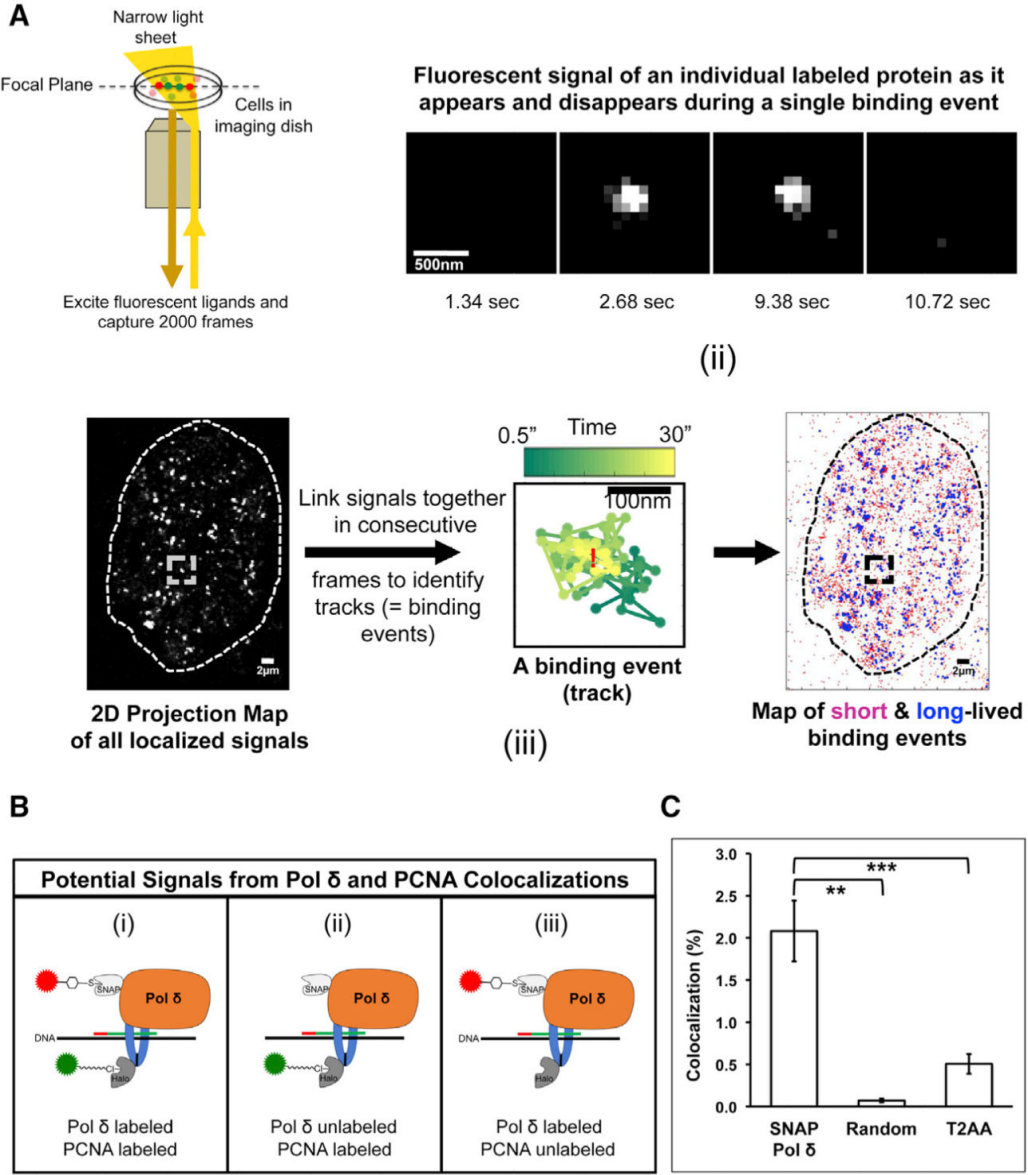
SMT in Live Cells by High-Resolution Microscopy Reveals Dynamic Interactions between Pol δ and PCNA That Are PIP Box Mediated (A) Schematic detailing live-cell SMT of fluorescently tagged Pol δ (p125) and PCNA. (i) Labeled cells in an imaging dish are illuminated by a laser focused to produce a narrow layer of illumination across the focal plane (HILO illumination); only labeled proteins within the focal plane are excited and fluoresce. The 2,000-frame time-lapse photomicrographic videos are captured while a high speed filter continuously alternates between SNAP and Halo dye channels to obtain near-simultaneous imaging of labeled SNAP- and Halo-tagged proteins. (ii) An image series of a single binding event. Frames have been skipped for simplicity; this event lasted from frame 3 s to 10.5 s. (iii) All of the signals for a given labeled protein seen within a nucleus (outlined) throughout an entire video capture are localized in a 2D map. Individual signals in consecutive frames located within a highly confined area (based on expected diffusion constants) are linked together to create a track, which identifies a binding event. The average position of the binding event (mean x and y-position) is indicated by a red X. Spatiotemporal information that defines a track can be used to classify binding events as short lived (<1.7 s, pink) or long lived (>1.7 s, purple). (B) Possible colocalization events involving genomic DNA-bound labeled Pol δ and/or labeled PCNA: (i) both SNAP-Pol δ and Halo-PCNA are labeled, (ii) only Halo-PCNA is labeled, and (iii) only SNAP-Pol δ is labeled. (C) Average colocalization rates of labeled SNAP-Pol δ and Halo-PCNA in untreated (SNAP-Pol δ), computer-randomized untreated (random), and T2AA-treated cells. LOX cells (C11) co-expressing SNAP-Pol δ and Halo-PCNA were pre-treated for 4 h with 20 μM T2AA, followed by dual-color SMT in the presence of 20 μM T2AA. A total of 59 nuclei from untreated cells and 49 nuclei from T2AA-treated cells from 4 independent experiments were analyzed. Computer-randomized values were obtained by randomizing the average positions of the same PCNA and Pol δ binding events analyzed from the 59 untreated cell nuclei. Student’s t test was used for statistical analysis. Error bars indicate SE. **p = 1.86 × 10^−7^, ***p = 0.0002.

Pol δ and PCNA work in concert at the replication fork during genome replication. To observe the dynamic interaction between these replication partners at the single-molecule level *in vivo*, we performed live-cell two-color SMT. LOX cells co-expressing tagged and fluorescently labeled Pol δ and PCNA were imaged using HILO (highly inclined and laminated optical sheet) illumination (Figure 2A) coupled with Motion-Blur microscopy (Chen et al., 2014). Using these conditions, single chromatin-bound protein molecules appear as sharp, single, diffraction-limited bright spots, whereas fast-diffusing unbound molecules appear as blurred signals. Each sharp fluorescent nuclear signal within the individual frames of a video imaging was then mapped by 2D Gaussian fitting. A chromatin binding event is identified as a signal track, where a given bright signal (molecule) is seen in multiple consecutive frames of a time-lapse video capture, localized within a highly confined area based on expected diffusion constants (Chen et al., 2014). This track establishes where and for how long a particular molecule was bound to the genome within the nucleus (Figure 2A). Importantly, we limited all of our SMT imaging and analysis to cells displaying a punctate distribution pattern of nuclear PCNA foci, defining them as S phase cells (Chagin et al., 2016). This allowed us to restrict our analysis to Pol δ and PCNA molecules primarily engaged in genome replication and not other cellular functions of these proteins, such as repair.

Based on abundant biochemical evidence, every Pol δ complex is presumably linked to a PCNA trimer when performing DNA synthesis. However, only a small amount of the total population of either PCNA or Pol δ p125 in our cells is tagged and fluorescently labeled due to sparse labeling required for high-resolution single-molecule imaging. Consequently, three types of labeled chromatin-bound Pol δ-PCNA complexes can be visualized (Figure 2B): (1) fluorescently labeled SNAP-Pol δ and Halo-PCNA bound to DNA at the same place and time, observed as a SNAP-Pol δ signal in close proximity of a Halo-PCNA track; (2) fluorescently labeled Halo-PCNA in complex with untagged/unlabeled Pol δ, which would appear as a signal of tagged PCNA only; and (3) untagged/unlabeled PCNA complexed with fluorescently labeled SNAP-Pol δ, which would appear as a signal of tagged Pol δ only (Figure 2B). Although all three types of complexes represent Pol δ-PCNA colocalization events, only complexes containing both fluorescently labeled Pol δ and PCNA (Figure 2Bi) can be definitively identified as Pol δ-PCNA colocalizations. Our analysis of the two-color SMT obtained for LOX cells expressing tagged and fluorescently labeled Pol δ and PCNA (Figure 1D) revealed that Halo-PCNA was colocalized (within <47 nm) with SNAP-Pol δ in 2.1% of the Pol δ tracks (Figure 2C; Videos S1 and S2). This level of observed colocalization is consistent with anticipated colocalization levels given the expression levels and required sparse labeling of the tagged proteins. In comparison, a computer randomization of the average positions of the same (experimentally observed) SNAP-Pol δ and Halo-PCNA tracks within the nucleus resulted in a colocalization frequency of 0.07% (Figure 2C).

The association of PCNA with its binding partners, including Pol δ, is generally mediated by interactions between PCNA and PIP box motifs found on binding partners (Choe and Moldovan, 2017; Slade, 2018). *In vitro* biochemical studies have shown that PCNA interaction with PIP-box-containing proteins could be disrupted by T2 amino alcohol (T2AA), a non-peptide small-molecule competitive inhibitor (Punchihewa et al., 2012). To establish that the Pol δ-PCNA colocalizations we detected by our SMT represented bona fide Pol δ p125-PCNA complexes tethered together by PIP box interactions, we tested if T2AA treatment could disrupt colocalizations of fluorescently labeled Pol δ and PCNA. Treatment of LOX C11 cells with T2AA followed by dual-color SMT revealed an ~4-fold reduction in colocalization (Figure 2C; and 2.1% to 0.5% of Pol δ tracks). These results indicate that the SNAP-Pol δ-Halo-PCNA colocalization events observed represent a Pol δ p125-PCNA complex, likely the Pol δ holoenzyme complex. Furthermore, our findings demonstrate that these complexes are held together by PCNA IDCL-Pol δ- PIP-box interactions *in vivo*.

### The Ordered Assembly and Disassembly of the Pol δ Holoenzyme on the Genome

**Figure 3.**
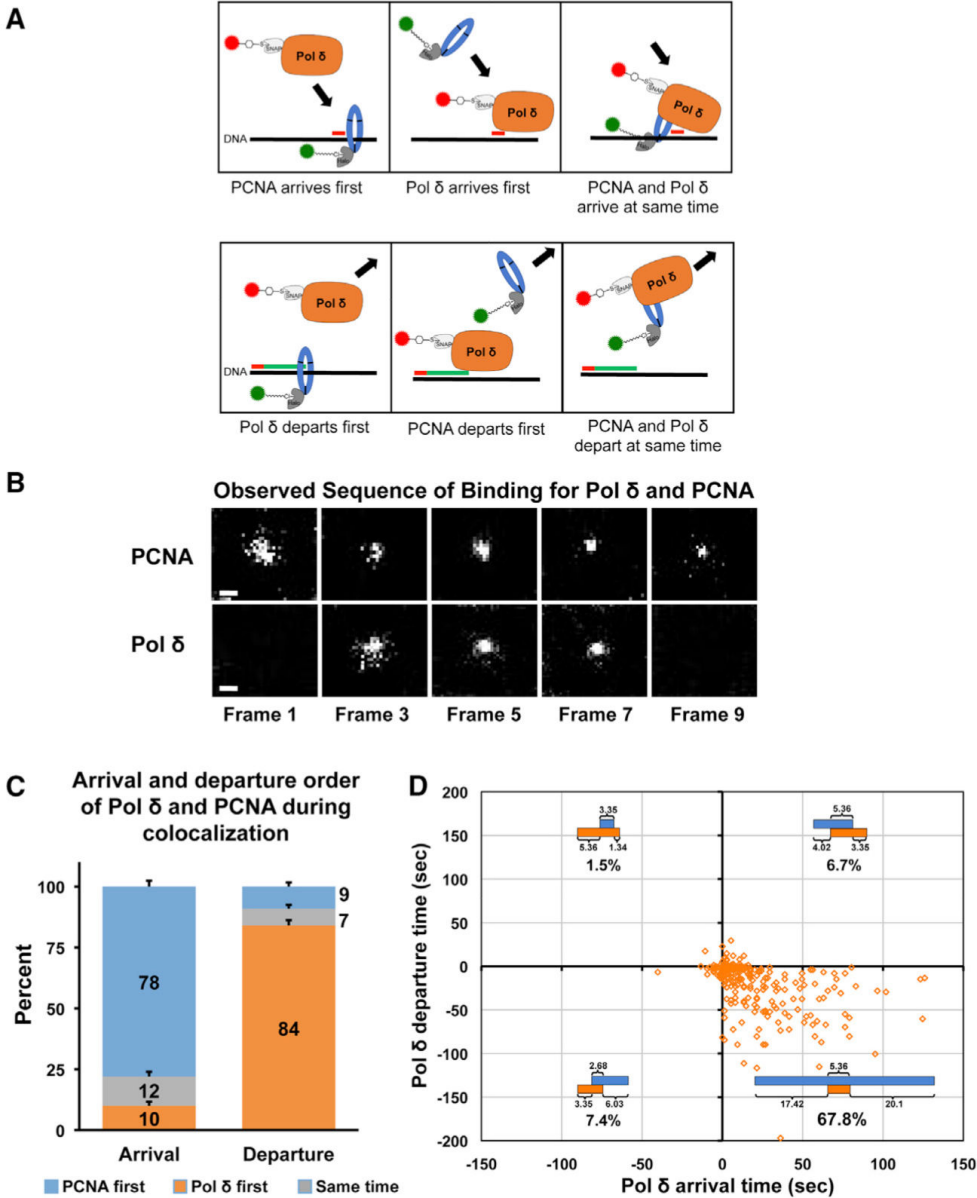
Pol δ Holoenzyme Assembly and Disassembly on the Genome Follow a Predominant Order (A) Potential order of SNAP-Pol δ and Halo-PCNA arrival and departure at sites of genome binding. Top: three sequence combinations in which Pol δ and PCNA can arrive on the primed DNA strand, namely, PCNA arrives first, Pol δ arrives first, or they both arrive simultaneously. Bottom: departure of Pol δ or PCNA could occur in one of the three ways, namely, Pol δ could depart first, PCNA could depart first, or the two could leave simultaneously. (B) An example of an image series of a typical colocalization binding event observed by dual-color SMT. In this particular event, colocalization occurred from frames 3–7 (~6-s duration). Note the Halo-PCNA signal appears before the SNAP-Pol δ signal and disappears after the Pol δ signal. Scale bars represent 500 nm. (C) Histogram of the arrival and departure order of SNAP-Pol δ and Halo-PCNA molecules during observed genomic colocalization events. The percentage of SNAP-Pol δ and Halo-PCNA molecules arriving and departing first or at the same time are indicated. Error bars indicate SD, as determined by Bootstrap analysis (1,000 iterations) of observed colocalizations. The predominant order of events observed is that PCNA loading precedes Pol δ binding, followed by Pol δ dissociation preceding PCNA unloading. (D) Scatterplot of SNAP-Pol δ arrival (binding) and departure (dissociation) relative to Halo-PCNA binding and dissociation during nuclear colocalization events. Timelines (blue and orange bars) shown in each quadrant indicate the binding order of SNAP-Pol δ and Halo-PCNA and time interval between arrival and departure of Pol δ/PCNA for colocalizations within that quadrant (SNAP-Pol δ arriving/departing before/ after Halo-PCNA). Blue bars indicate the presence of genome-bound Halo-PCNA during colocalization events; orange bars indicate the presence of genome-bound SNAP-Pol δ (p125) during colocalization events. Median time intervals (s) between arrival (before colocalization) and departure (after colocalization) of PCNA/Pol δ are shown below bars; median time interval (s) of colocalization shown above bars. The 95% confidence interval of median times is reported in Figure S2. Percentage of total colocalizations represented by the specific order of assembly/disassembly in each quadrant is indicated under timelines. Plots reveal multiple assembly/disassembly pathways of the holoenzyme. A total of 270 colocalization events from 59 nuclei of LOX C11 cells from 4 independent experiments were analyzed.

The assembly of the Pol δ holoenzyme complex on chromatin is thought to be initiated by loading of the PCNA homotrimer ring onto primed DNA at the DNA template/RNA primer 3′ end junction, the site where DNA synthesis begins. Pol δ (δ4 or δ3) then binds to the loaded PCNA ring to form the holoenzyme complex. To establish if this is indeed how the holoenzyme assembles *in vivo*, we analyzed the SNAP-Pol δ and Halo-PCNA tracks where we saw colocalization and determined the specific video frame numbers in which each colocalized track appeared, colocalized, and disappeared to reconstruct the binding order of SNAP-Pol δ and Halo-PCNA. There are three sequence combinations in which Pol δ and PCNA can arrive on the primed DNA strand: PCNA arrives first, Pol δ arrives first, or they both arrive simultaneously (Figure 3A, top). An example of a typical binding event image series is shown in Figure 3B. By analyzing 270 colocalization events from 59 cell nuclei, we observed that Halo-PCNA predominantly arrived first, followed by SNAP-Pol δ (Figure 3C). The majority (~79%) of SNAP-Pol δ molecules arrived after PCNA, generally from ~1 to 30 s after PCNA loading (Figure S1, top left). Upon SNAP-Pol δ binding, the two molecules colocalized for a median time of ~4 s. The order of departure of PCNA and Pol δ could occur one of the three ways: Pol δ could depart first, PCNA could depart first, or the two could leave simultaneously (Figure 3A, bottom). We found that SNAP-Pol δ predominantly (~84% of colocalized molecules) departed before Halo-PCNA (Figure 3C). After departure of SNAP-Pol δ, Halo-PCNA remained on chromatin for ~1 to 30 s (Figure S1, bottom left). It should be noted that when PCNA was bound to chromatin for a much longer time than it was colocalized with Pol δ, it is possible that unlabeled Pol δ could be replacing labeled Pol δ prior to PCNA dissociation, given the underlabeling of Pol δ. Further analysis SNAP-Pol δ arrival (binding) and departure (dissociation) timing relative to Halo-PCNA binding and dissociation of individual colocalization events revealed multiple assembly/disassembly pathways of the holoenzyme (Figure 3D). We found that PCNA loading, followed by Pol δ binding, followed by Pol δ unloading, and then PCNA dissociation constituted the main assembly/disassembly pathway, occurring in ~68% of all colocalizations (Figure 3D, bottom right quadrant). One key feature we observed in this pathway was the long, stable binding of PCNA compared to PCNA binding in the other alternate pathways. We also found that loading order had a distinct effect on complex stability. In pathways where Pol δ loading preceded PCNA loading (Figure 3D, top and bottom left quadrants), we observed a reduction in the amount of time Pol δ and PCNA colocalized compared to when PCNA loaded first (Figure 3D, top and bottom right quadrants). Collectively, our findings reveal that Pol δ holoenzyme assembly and disassembly *in vivo* are ordered and follow a predominant pathway, where PCNA loading precedes Pol δ binding, followed by Pol δ dissociation preceding PCNA unloading.

### Pol δ’s Enzymatic Activity Regulates the Assembly and Disassembly Dynamics of the Holoenzyme

**Figure 4.**
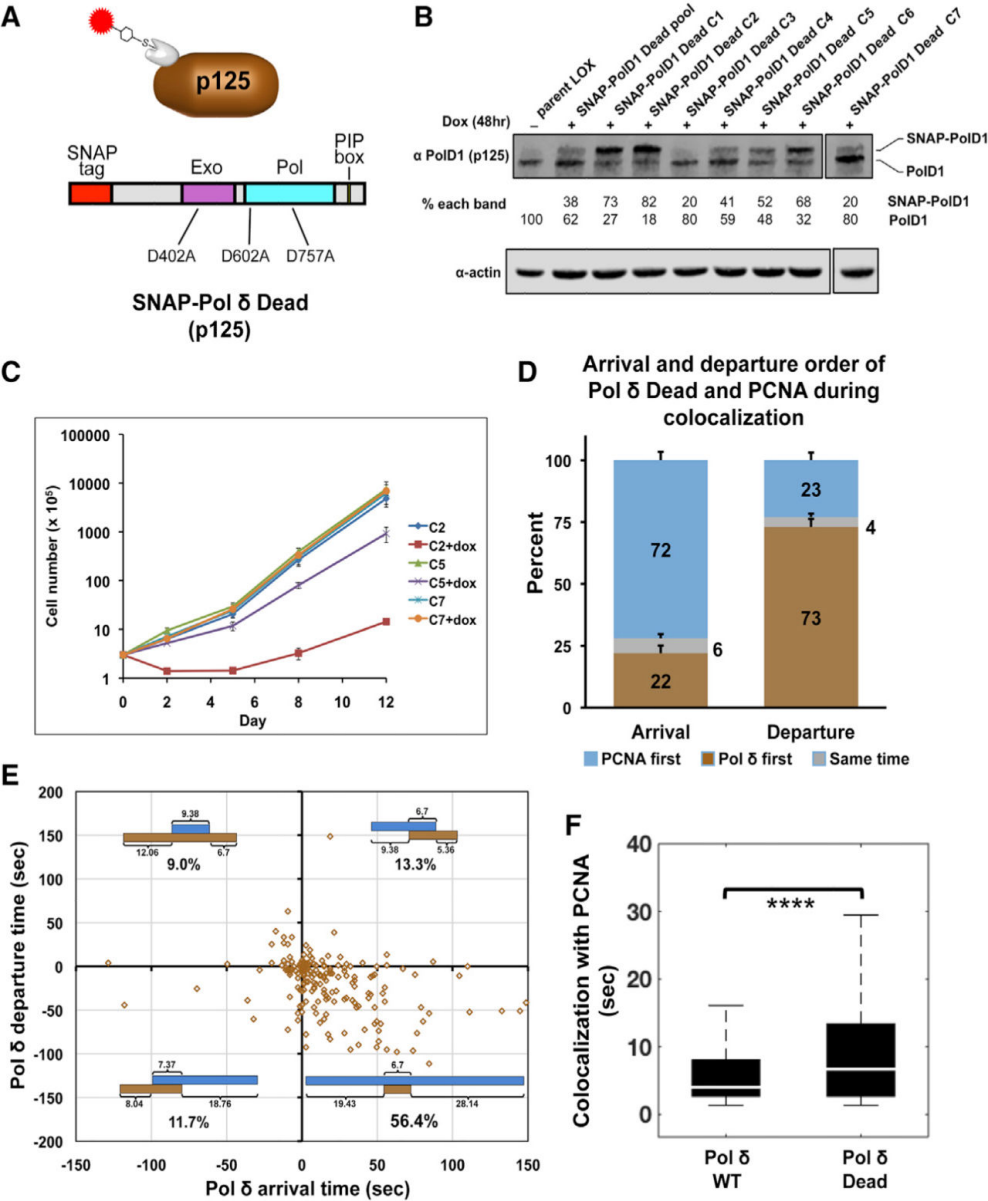
Pol δ’s Enzymatic Activity Regulates the Assembly and Disassembly Dynamics of the Holoenzyme (A) Schematic diagram of SNAP-Pol δ Dead p125 fusion protein (PIP, PCNA interacting protein). In this mutant, two essential active site residues that catalyze DNA synthesis (Asp602 and Asp757) and a residue essential for the 3′ –5′ exonuclease proofreading activity of Pol δ (Asp402) were mutated to alanine, resulting in a catalytically dead protein. (B) Immunoblot of cell lysates of LOX cell lines stably expressing doxycycline-inducible SNAP-Pol δ Dead p125. The percentage of the total Pol δ p125 protein represented by SNAP-tagged (top band) and untagged endogenous (bottom band) proteins is shown below each lane. (C) Growth curve of LOX stable cell lines co-expressing SNAP-Pol δ Dead p125 and Halo-PCNA proteins. Cultures of three clonal lines, C2, C5, and C7 (82%, 52%m and 20% of total Pol δ p125 is SNAP-tagged, respectively), were grown in the absence or presence (+ dox) of 1 μg/ml doxycycline and cells counted at time points indicated (cell number is relative to initial seeding). Values were obtained from two independent cultures. Error bars indicate SD. (D) Histogram of the arrival and departure order of SNAP-Pol δ and Halo-PCNA molecules during 188 observed nuclear colocalization events (from SNAP-Pol δ Dead line C2). The percentage of SNAP-Pol δ and Halo-PCNA molecules arriving and departing first or at the same time are indicated. Error bars indicate SD as determined by Bootstrap analysis (1,000 iterations) of observed colocalizations. A 2-fold greater percentage of SNAP-Pol δ Dead molecules arrive before Halo-PCNA and depart after PCNA than SNAP-Pol δ WT. (E) Scatterplots of SNAP-Pol δ arrival (binding) and departure (dissociation) relative to Halo-PCNA binding and dissociation during nuclear colocalization events. Timelines (blue and orange-brown bars) shown in each quadrant indicate the binding order of SNAP-Pol δ and Halo-PCNA and time interval between arrival and departure of Pol δ/PCNA, as described in Figure 3. The 95% confidence interval of median times is reported in Figure S2. (F) Boxplots comparing the duration of Halo-PCNA colocalizations with SNAP-Pol δ WT (C11) versus SNAP-Pol δ Dead (C2). A total of 59 nuclei from SNAP-Pol δ WT cells from 4 independent experiments and 13 nuclei from SNAP-Pol δ Dead cells from 2 independent experiments were analyzed for colocalization. A Kruskal-Wallis test was used to determine pairwise significance. ****p = 2.21 × 10^−6^.

The interaction of PCNA with Pol δ greatly stimulates processive DNA synthesis by the polymerase by enhancing stable binding of the enzyme to DNA. However, it is unknown whether polymerase activity affects the stability of the bound PCNA-Pol δ holoenzyme complex. To examine the effect of polymerase activity on binding dynamics, we generated a catalytically inactive SNAP-Pol δ mutant (SNAP-Pol δ Dead), where the DNA synthesis and 3′–5′ exonuclease activities were inactivated (Figure 4A). This mutant was stably and inducibly co-expressed in the Halo-PCNA-expressing LOX cell line (Figure 1C). When p125 levels in these cells were examined, we found a similar modulation of p125 expression, as was seen with the wild-type (WT) SNAP-Pol δ (i.e., endogenous p125 expression was inversely proportional to SNAP-Pol δ Dead expression) (Figure 4B). Importantly, unlike expression of the WT tagged protein, SNAP-Pol δ Dead expression led to retardation of cell growth that was tightly correlated to the level of the dead mutant, i.e., growth rates decreased as expression of the mutant protein increased (Figure 4C). Notably, this further indicated that the SNAP-tagged protein can substitute for the endogenous protein in Pol δ complexes assembled *in vivo*.

Dual-color SMT of LOX cells co-expressing SNAP-Pol δ Dead and Halo-PCNA (Figure 4B; Videos S3 and S4) revealed a significant difference in holoenzyme assembly of the mutant compared to the WT Pol δ. Specifically, there was an increase in the number of colocalization events where Pol δ loads onto the genome before PCNA. In approximately 22% of the 188 detected colocalization events, Pol δ Dead arrived before PCNA (Figures 4D and S1, top right). Comparatively, SNAP-Pol δ WT arrived before Halo-PCNA only ~10% of the time (Figures 3C and S1, top left). There was little difference in the percent of SNAP-Pol δ Dead and Halo-PCNA molecules that colocalized (out of total SNAP-Pol δ Dead tracks) compared to SNAP-Pol δ WT track colocalizations (data not shown). In addition, when we examined the order in which molecules departed, we found there was a >2-fold increase in the percentage of Pol δ remaining bound after Halo-PCNA departed (~23% SNAP-Pol δ Dead versus ~9% SNAP-Pol δ WT; Figures 3C, 4D, and S1). Analysis of SNAP-Pol δ arrival and departure timing relative to Halo-PCNA binding and dissociation of individual colocalization events (Figure 4E) revealed further differences between WT and Dead proteins. We found a reduction in colocalizations occurring by the main assembly/disassembly pathway (PCNA loading/Pol δ loading/colocalization/Pol δ unloading/PCNA unloading) from ~68% (WT) to ~56% (Dead) (compare Figure 3D, bottom right quadrant, and Figure 4E, bottom right quadrant). We also found that, similar to with SNAP-Pol δ WT, loading order had a distinct effect on Pol δ-Dead-PCNA complex stability. However, in contrast to Pol δ WT, in pathways where Pol δ Dead loading preceded PCNA loading (Figure 4E, top and bottom left quadrants), we observed an increase in the amount of time Pol δ and PCNA colocalized compared to when PCNA loaded first (Figure 4E, top and bottom right quadrants). In general, once both proteins were bound, we found that Halo-PCNA had a longer duration of colocalization with the catalytically dead polymerase than with the tagged WT polymerase (Figure 4F; median colocalization time of ~7 s Dead versus ~4 s WT). It is unlikely that photobleaching had any significant effect on the measurement of release time and dissociation order. If it did, we would not observe different WT and Dead Pol δ release times (compare Figures 3D and 4E). This is because photobleaching is primarily a property of the dye and is not affected by the protein attached to the dye. Thus, labeled WT and Dead Pol δ would be expected to have the same apparent release times if photobleaching were being interpreted as protein release. Moreover, the initial binding order would not be affected by photobleaching. Collectively, these data suggest that once loaded onto chromatin, the dead polymerase is more stably associated than the WT polymerase, even in the absence of the PCNA sliding clamp.

### The Catalytic Activity of Pol δ Affects Its Residence Time on Chromatin and the Percentage of Molecules That Bind for Longer Periods

**Figure 5.**
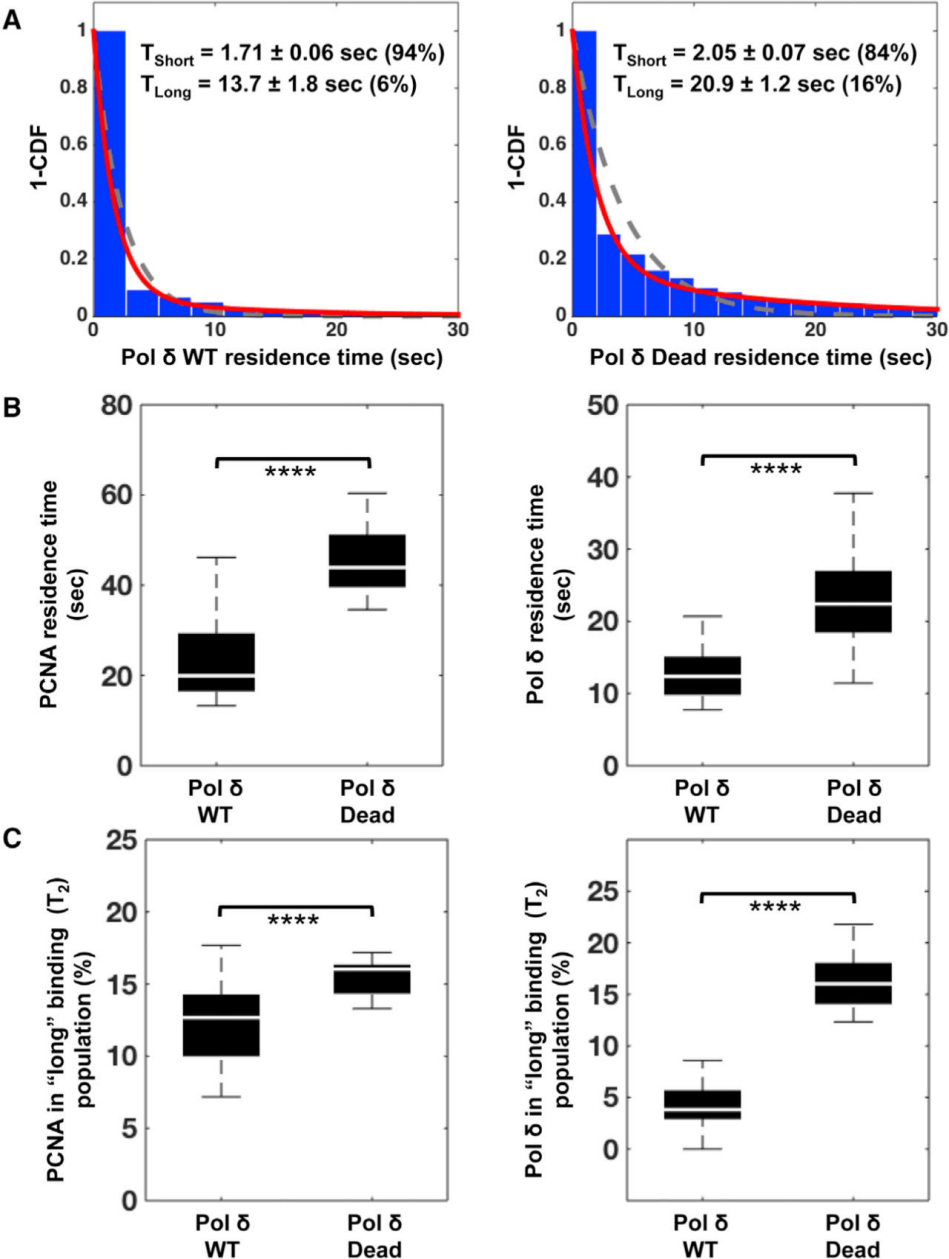
The Catalytic Activity of Pol δ Affects Its Residence Time on Chromatin (A) Residence times were determined from 1–cumulative distribution function (1–CDF) plots of nuclear SNAP-Pol δ WT and Dead bound to chromatin (representative examples shown) fitted to a single (gray dashed) or two-component (red solid) exponential decay model. These curves identified two predominant populations: one of short (<1.7 s) unstable binding events (T_short_ = T_1_ population) and one of long (>1.7 s) stable binding events (T_long_ = T_2_ population). Percentage of total binding events in each population indicated in parentheses. (B) Boxplot comparison of genome residence times for SNAP-Pol δ WT, SNAP-Pol δ Dead, and Halo-PCNA. Left: residence time of Halo-PCNA when co-expressed with either SNAP-Pol δ WT or SNAP-Pol δ Dead. Residence times for Halo-PCNA were determined from 1–CDF plots of nuclear Halo-PCNA bound to chromatin, as described above for SNAP-Pol δ (****p = 4.94 × 10^−7^). Right: residence time of SNAP-Pol δ WT and SNAP-Pol δ Dead when co-expressed with Halo-PCNA (****p = 1.62 × 10^−5^). (C) Boxplot comparison of the percent of total genome binding events in the “long” T_2_ binding population for SNAP-Pol δ WT, SNAP-Pol δ Dead, and Halo-PCNA. Left: percentage of Halo-PCNA in T_2_ when co-expressed with either SNAP-Pol δ WT or SNAP-Pol δ Dead (****p = 5.50 × 10^−5^). Right: percentage of SNAP-Pol δ WT or SNAP-Pol δ Dead in T_2_ when co-expressed with Halo-PCNA (****p = 1.97 × 10^−8^). Binding events from 59 nuclei of SNAP-Pol δ WT (C11) cells from 4 independent experiments and 13 nuclei of SNAP-Pol δ Dead (C2) cells from 2 independent experiments were analyzed. A Kruskal-Wallis test was used to determine pairwise significance.

One key difference we saw between the behavior of the WT and catalytically inactive Pol δ complexes was that Pol δ Dead colocalized with Halo-PCNA for longer periods of time (Figure 4F). This suggested that the catalytic activity of the polymerase might be affecting its chromatin binding dynamics, which should be reflected in its residence time on chromatin. Therefore, we compared residence times of SNAP-Pol δ WT, SNAP-Pol δ Dead, and Halo-PCNA tracks imaged from LOX cells co-expressing Halo-PCNA with either SNAP-Pol δ WT or SNAP-Pol δ Dead. Custom MATLAB scripts were used to bin and plot all the tracks according to residence time (T), i.e., the duration of the binding event, in 1-s intervals. Single and double exponential decay curves were fit to the binned data (Figure 5A), with best fits obtained with the double exponential models, which identified two distinct predominant populations within the plotted data. One population, designated T_1_, represented tracks that had a relatively “short” residence time, typically 2 s or less. The T_1_ population comprises most of the tracks, 84%–96% of the total depending on the tagged protein (Figures 5A and S3). The second population, designated T_2_, had relatively “long” residence times, greater than 2 s, which represented 4% to 16% of total tracks (Figures 5A and S3). We consider these tracks stable binding events and, in the case of SNAP-Pol δ WT, events likely to be mostly associated with productive DNA synthesis.

When we compared residence times of the tagged proteins, we saw an increase in residence time of the stable binding population (T_2_) for both SNAP-Pol δ and Halo-PCNA when Halo-PCNA was co-expressed with Pol δ Dead versus Pol δ WT. PCNA residence time of the stable population increased from 20 s to 43.9 s (Figures 5B and S2), whereas SNAP-Pol δ residence time increased from 12.4 s (Pol δ WT) to 22.4 s (Pol δ Dead) (Figures 5A, 5B, and S3). Similarly, we also observed an increase in the percentage of molecules in the T_2_ population, the long stable binders. The SNAP-Pol δ T_2_ population increased from 4% (Pol δ WT) to 16% (Pol δ Dead) (Figures 5C and S3). The increases in both residence time and the size of the T_2_ population indicate that not only did the tracks last for longer periods but also the proportion of tracks lasting longer also increased. These increases likely reflect the difference in the activity of the T_2_ stable binding populations, the WT T_2_ containing enzymatically active polymerases, whereas the Dead T_2_ consists of inactive Pol δ. These findings further suggest that the dead polymerase is more stably associated than the WT polymerase once loaded onto chromatin.

### Pol δ Forms Foci of Binding Events That Are Spatiotemporally Related

**Figure 6.**
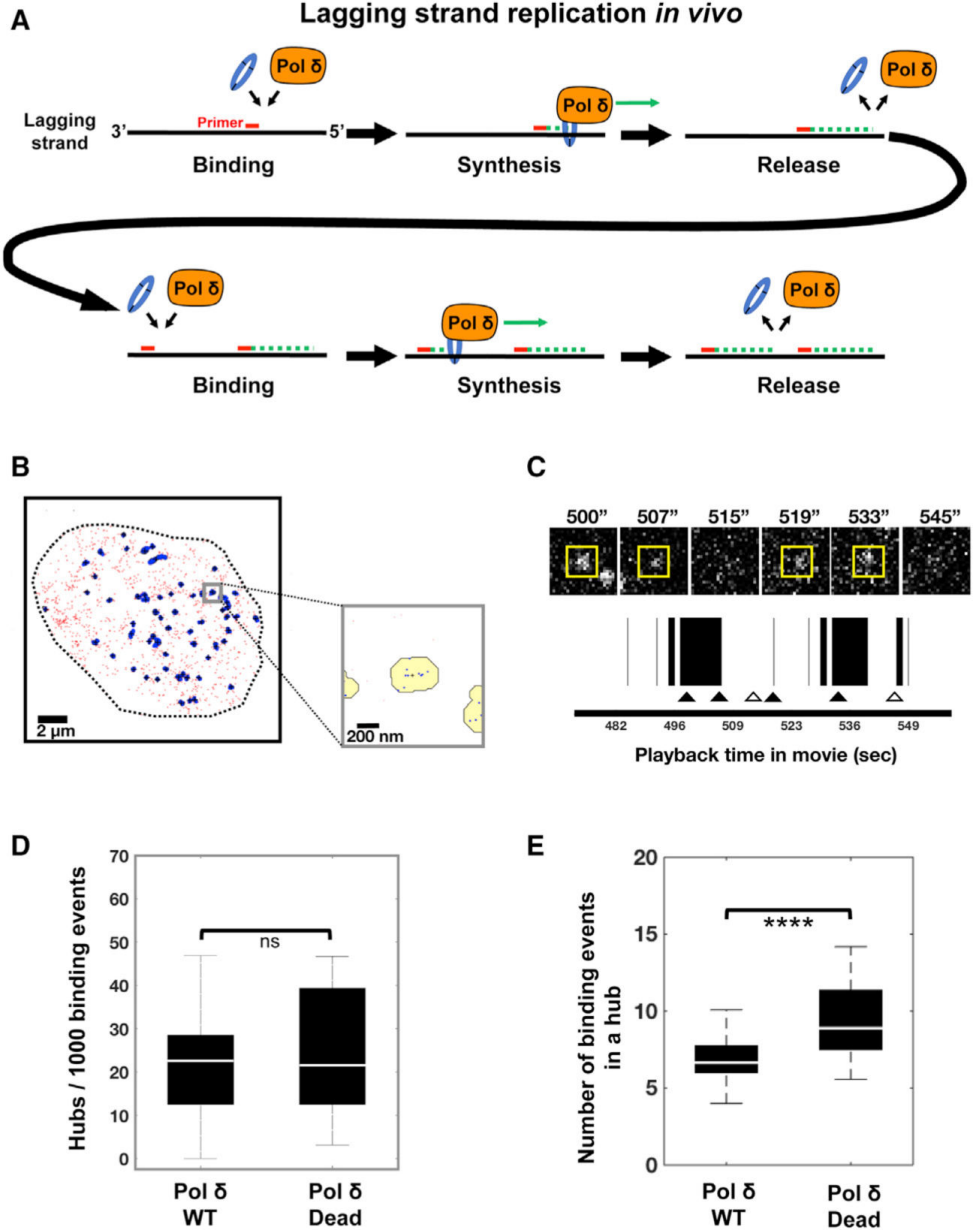
Pol δ Forms Foci of Binding Events That Are Spatiotemporally Related (A) Discontinuous lagging strand replication involves cycles of Pol δ binding synthesis release and Pol δ revisiting. (B) Global 2D map of WT Pol δ nuclear binding events shown at left. Chromatin binding events lasting longer than 1.7 s are shown (red dots). Clustering analysis algorithms revealed repeated Pol δ binding events within small foci (hubs) shaded in blue. Expanded inset (bottom right) of boxed region in left panel illustrates clustering of Pol δ binding events (blue dots) within hubs shaded in yellow (with black outline). (C) Close-up image series of a single hub (yellow box) during cycles of revisiting by WT Pol δ shown in top panel. A playback timeline of binding events is shown below image series. Black bars indicate bound Pol δ. Bar width corresponds to duration of binding event (when Pol δ appears then disappears). (D) Comparison of the number of Pol δ hubs per 1,000 binding events for SNAP-Pol δ WT or SNAP-Pol δ Dead. (E) Comparison of the number of Pol δ binding events per hub for SNAP-Pol δ WT or SNAP-Pol δ Dead (****p = 0.001). Binding events from 59 nuclei of SNAP-Pol δ WT (C11) cells from 4 independent experiments and 13 nuclei of SNAP-Pol δ Dead (C2) cells from 2 independent experiments were analyzed. A Kruskal-Wallis test was used to determine pairwise significance.

Lagging strand copying is a primary function of Pol δ. Because it occurs in a discontinuous manner involving cycles of Pol δ binding synthesis dissociation (Figure 6A), Pol δ is expected to revisit sites of active lagging strand copying. Therefore, we analyzed SNAP-Pol δ genome binding events to determine if revisiting was seen. We performed high-resolution clustering analysis to identify small foci (~250 nm) of repeated Pol δ binding events (Figure 6B). This analysis revealed the presence of distinct nuclear foci or hubs where Pol δ dynamically arrives and departs on the genome repeatedly (Figures 6C and 6D). The median number of these hubs was the same for both Pol δ WT and Pol δ Dead, approximately 22 hubs per 1,000 Pol δ binding events (Figure 6D). However, a comparison of the WT and Dead hubs (Figure 6E) showed that Dead hubs contained 33% more binding events per hub, indicating that Pol δ Dead revisited the same site more frequently than WT Pol δ. We further analyzed the hubs to determine the latency of Pol δ binding in hubs (i.e., the time interval between Pol δ revisits in a hub). A histogram of the time intervals between Pol δ revisits was fit with a single-, double-, and triple-component exponential decay model (Figure S4A). We found that the best fits were obtained with the triple exponential model, which identified three distinct predominant populations of latency times. These consisted of short (~6–10 s), intermediate (~23–27 s), and long (~160–230 s) time intervals (Figure S4B). A comparison of the percentages of each population revealed that Pol δ Dead showed a higher percentage (50% versus 37%) of short latency times between hub binding events (Figure S4C). This indicated that the dead polymerase more rapidly revisits a hub. Notably, because WT and Dead Pol δ displayed different latency profiles, it is unlikely that dye blinking of bound SNAP-Pol δ significantly contributes to measurements of revisiting. If the observed revisiting events were mainly blinking events, then we would not see differences in the revisiting time profiles between the WT and Dead Pol δs. This is because dye blinking is primarily a property of the dye, not the labeled protein, so labeled WT and Dead Pol δ would exhibit the same blinking and, therefore, the same apparent time between revisits. Importantly, because the Pol δ Dead displayed more frequent and rapid hub revisiting than the WT, this suggests that the replisome recognizes the lack of synthesis by the dead polymerase and the need to replace Pol δ at that site, Taken together, our clustering analysis indicates that human Pol δ is frequently replaced at replication sites and suggests that these binding hubs represent bona fide regions of lagging strand synthesis.

## DISCUSSION

Pol δ performs essential roles in genome integrity maintenance, including serving as the main polymerase tasked with lagging strand DNA synthesis during genomic replication. Using stably expressed fluorescently tagged Pol δ and PCNA, we used SMT to study the dynamics of Pol δ interaction with chromatin and PCNA in real time in living human cells. Our *in vivo* investigations reveal key aspects of Pol δ chromatin binding behavior, Pol δ-PCNA holoenzyme assembly/disassembly pathways, and Pol δ p125 catalytic subunit protein regulation.

Pol δ was tracked in our experiments by a SNAP-tagged p125 catalytic subunit. Uncomplexed, free p125 is essentially unable to stably bind primed DNA templates, even in the presence of PCNA (Zhou et al., 2012), and Pol δ complexes have extremely low DNA binding affinities in the absence of PCNA (Hedglin et al., 2016). Therefore, the chromatin-bound p125 molecules we detected are likely part of active Pol δ-PCNA holoenzyme complexes. Our cell culture results also indicate that the tagged p125 is incorporated into a functional Pol δ holoenzyme based on normal growth rates observed when >90% of endogenous cellular p125 is replaced by SNAP-Pol δ p125 (Figure 1E). Furthermore, the inhibition of SNAP-Pol δ (p125)-Halo-PCNA colocalization events by the PIP box inhibitor T2AA strongly suggests that the two are forming authentic Pol δ holoenzyme complexes held together by PIP-box-mediated interactions.

Our present studies revealed that the majority of Pol δ binding interactions with chromatin are brief (<1.7 s), suggesting that the polymerase is constantly scanning the genome for target replication sites. These interactions may be indicative of the polymerase searching for a primer-template-bound PCNA ring. Alternatively, these short-lived binding events may represent fast polymerase turnover similar to the rapid concentration-dependent exchange seen within the *Escherichia coli* replisome by Pol III (Lewis et al., 2017) and in the *Bacillus subtilis* replisome by Pol C (Liao et al., 2016). *In vitro* data suggest fast equilibrium kinetics between the formation and disassembly of the human Pol δ replicative complex on DNA templates (Hu et al., 2012; Masuda et al., 2007; Podust et al., 2002). However, the bacterial polymerase binding events last ~3–4 s during rapid exchanges, longer than the brief Pol δ interactions. We also observed less frequent, substantially longer stable chromatin binding events that lasted ~13 s, which presumably represented a subpopulation of Pol δ (δ3 or δ4) complexes engaged in productive DNA synthesis. In the context of lagging strand synthesis, *in vitro* estimates indicate that complete Okazaki fragments (100–250 nt) could be synthesized by human Pol δ4 in 1–2.5 s, based on a k_*pol*_ (polymerization rate constant) of 87–108 nt sec^−1^ (Hedglin et al., 2016; Meng et al., 2010) or by Pol δ3 (k_*pol*_ = ~20 nt sec^−1^) (Meng et al., 2010) in 5–12 s. These time frames correlate well with the *in vivo* average chromatin residence times we observe for the short (T_1_) and long binding (T_2_) populations of Pol δ (Figure S3). Alternatively, these different populations may represent polymerases engaged in copying of genomic regions of different degrees of secondary structure. It has been shown that Pol δ holoenzyme polymerization rate varies greatly according to DNA sequence, slowing significantly within non-B DNA (Shah et al., 2010; Walsh et al., 2013). In addition to template copying, it is likely some of the Pol δ signals we detect are polymerase molecules involved in the Okazaki fragment maturation. During this process, Pol δ and PCNA, in complex with FEN1, sequentially remove RNA primers by nick translation (Stodola and Burgers, 2016). Accordingly, it is expected that a small percentage of the Pol δ binding events would last longer than 13 s, which we also observe in live cells. Similarly, some of the of the longer lived PCNA binding events we detect may represent PCNA trimers involved in Okazaki fragment maturation. Moreover, some longer lived PCNA binding events may be PCNA participating in other Pol δ-independent aspects of DNA replication. These include facilitating bypass of replication-blocking lesions, protection of stalled replication forks from collapse, fork restart by fork reversal, and Okazaki fragment ligation (Choe and Moldovan, 2017; Slade, 2018).

Current understanding of human Pol δ holoenzyme assembly and disassembly dynamics has been largely based on *in vitro* examination of the individual steps of Pol δ loading and PCNA loading/unloading in isolation. This has led to models where the holoenzyme is constructed on a primed DNA template by the stepwise, ordered addition of PCNA (by the RFC clamp loader) followed by Pol δ (Hedglin et al., 2013). By inference, holoenzyme disassembly has been thought to occur in the reverse order.

**Figure 7.**
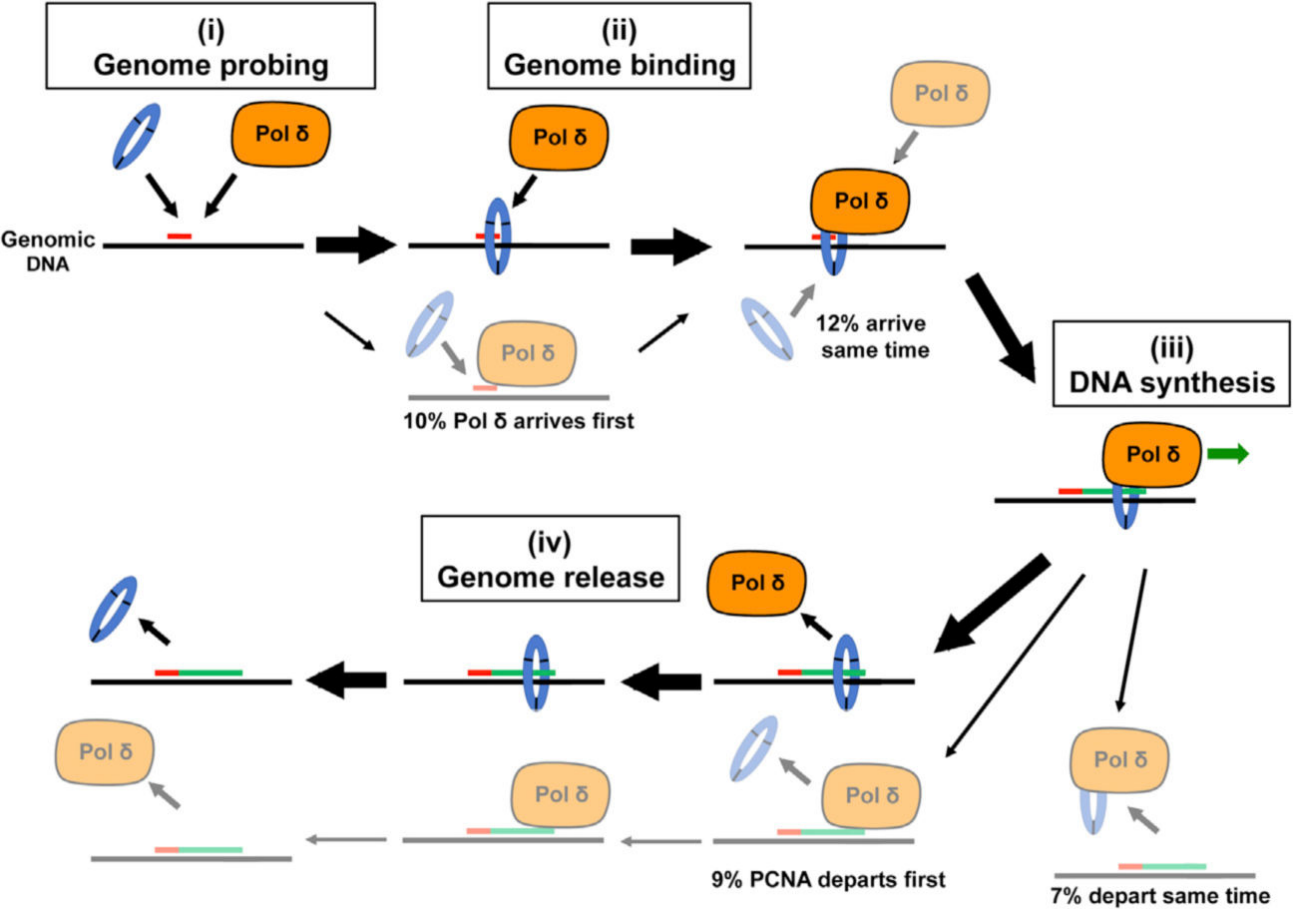
Pol δ Holoenzyme Assembly and Disassembly *In Vivo* Pathways of Pol δ holoenzyme assembly and disassembly *in vivo* revealed by SMT. (i) Pol δ and PCNA actively probe genome for target replication sites; (ii) stable genome binding by Pol δ and PCNA can occur sequentially or simultaneously, resulting in holoenzyme assembly; (iii) DNA synthesis by the assembled holoenzyme; and (iv) genome release by Pol δ and PCNA can occur sequentially or simultaneously. Bold arrows indicate the predominant assembly/disassembly pathway utilized, where PCNA loading precedes Pol δ binding followed by Pol δ dissociation preceding PCNA release. Alternate pathways are indicated by lighter images; percentage of alternate pathway usage (reported in Figure 3C) is indicated.

Evidence from biochemical studies on yeast Pol δ showed Pol δ disassembles from DNA templates before PCNA (Langston and O’Donnell, 2008). However, yeast Pol δ disassembly required a collision release mechanism, where polymerizing Pol δ dissociates from PCNA upon collision with the end of a downstream DNA duplex, and we are not aware of evidence that such a mechanism is utilized by human Pol δ. Moreover, the nuclear environment is very complex, with extensive interplay between all of the replication and chromatin-bound factors present in living nuclei. Recent studies have identified approximately 350 proteins that display phenotypes consistent with activities at replication forks or nascent chromatin (Wessel et al., 2019). Therefore, it is necessary to study holoenzyme behavior in the physiological setting to fully understand holoenzyme biology in the living system. *In vivo* PCNA binding dynamics have been examined by fluorescence recovery after photobleaching (FRAP) (Essers et al., 2005), although its behavior as part of the Pol δ holoenzyme complex in the context of *in vivo* DNA replication has yet to be studied. Importantly, our real-time SMT studies of Pol δ-PCNA colocalization events conclusively reveal the actual pathways of holoenzyme assembly and disassembly in living cells. We found that PCNA most frequently bound to the genome before Pol δ loaded and dissociated from chromatin after Pol δ unloaded (Figure 7).

In addition to the predominant holoenzyme assembly/disassembly pathway, we also observed alternate pathways (Figure 7). Collectively these alternative pathways constitute over 15% of WT events (Figure 3D) and 34% of dead polymerase events (Figure 4E). These included instances where Pol δ loaded onto chromatin before PCNA as well as examples of Pol δ dissociating from chromatin after PCNA (Figures 3 and 4). This was somewhat unexpected, as Pol δ complexes display low DNA binding affinities *in vitro* (Hedglin et al., 2016). This may indicate that there are additional chromatin-associated factors that increase the DNA binding stability of Pol δ complexes. Alternatively, considering the sparse labeling of PCNA, instances where Pol δ appears to load before PCNA may represent labeled Pol δ associated with bound unlabeled PCNA being recycled/exchanged to newly bound, labeled PCNA. Alternative pathways where PCNA is released before Pol δ may be used when Pol δ processivity and DNA binding affinity needs to be reduced, such as when the polymerase must release at the end of a nascent Okazaki fragment. Furthermore, occurrences of Pol δ unloading from chromatin after PCNA could reflect holoenzyme stalling/obstruction, especially in difficult-to-replicate sites of the genome. This is consistent with the increase in these events that we observe when Pol δ Dead, which is essentially a “stalled” polymerase, is substituted for Pol δ WT (compare Figures 3C, 3D, 4D, and 4E). Thus, PCNA may need to unload first in order for the stalled polymerase to be cleared from the fork and for copying to resume. Lending support to this concept, *in vitro* evidence indicates that translesion synthesis polymerases can exchange with Pol δ stalled at repetitive elements to continue template copying, by a PCNA-independent process (Barnes et al., 2017).

Human Pol δ becomes a significantly more processive polymerase when assisted by PCNA. The estimated *in vitro* processivity values of PCNA-Pol δ4 and PCNA-Pol δ3, derived from the ratio of k_*pol*_ to k_*cat*_ (turnover number), are 350 and 106 nt, respectively (Lee et al., 2017; Meng et al., 2010). However, processivity assays with PCNA-Pol δ4 indicate that a substantial portion (~14%–31%) of Pol δ may dissociate from the lagging strand before completing synthesis of a given Okazaki fragment (Hedglin et al., 2016). This implies more than one Pol δ may participate in the synthesis of an individual Okazaki fragment.

Our clustering analysis of Pol δ binding events demonstrates revisiting of Pol δ to specific sites (Figure 6). This may be indicative of successive Okazaki fragments being synthesized within a replication hub. However, we found genome-bound Halo-PCNA was often present for up to 20 s before SNAP-Pol δ arrived and up to 30 s after Pol δ left (Figures 3, 4, and S1). Therefore, PCNA was generally bound for significantly longer than the length of most of the observed colocalization events (Figure 4F). Considering that substantial unlabeled Pol δ is also present in the cell, it is possible that unlabeled Pol δ is exchanging with labeled Pol δ in the interval between PCNA loading and unloading. Our Halo-PCNA-SNAP-Pol δ colocalization results further suggest multiple Pol δ binding events may be needed to synthesize an Okazaki fragment *in vivo*. We found Halo-PCNA-SNAP-Pol δ colocalizations lasted for a median time of ~4s (Figure 4F), enough time for Pol δ4 to synthesize ~425 nt and for Pol δ3 to synthesize ~80 nt, based on *in vitro* rates (Hedglin et al., 2016; Meng et al., 2010). However, the Pol δ holoenzyme polymerization rate is greatly influenced by template sequence, slowing particularly through repetitive elements (Shah et al., 2010; Walsh et al., 2013), which comprise approximately 50% of the human genome (Treangen and Salzberg, 2011). Therefore, it would be predicted from processivity and polymerase rate measurements that several Pol δ3 (or possibly δ4) molecules would be required to synthesize of an Okazaki fragment. Furthermore, it has been suggested that the Pol δ3 complex is the default lagging strand polymerase despite having lower polymerase activity than the δ4 assembly. This is because the δ3 assembly has higher fidelity than the δ4 complex, has lower strand displacement activity than δ4, and, therefore, is less capable of generating undesirable, harder to process long flaps on Okazaki fragments (Lee et al., 2017). Thus, if Pol δ3 is the primary lagging strand polymerase *in vivo*, our results are consistent with multiple Pol δ molecules participating in synthesizing a single Okazaki fragment.

Interestingly, the loss of Pol δ catalytic activity led to distinct changes in the chromatin and PCNA binding dynamics of the enzyme. SNAP-Pol δ Dead complexes displayed increased chromatin residence times and longer Halo-PCNA colocalization times. In comparison to the active enzyme, Pol δ Dead also more frequently remained bound to chromatin after colocalized PCNA unloaded and for longer times. These increases likely reflect the time required for the replisome to sense the lack of polymerization and the need to clear the inactive polymerase from the fork. Importantly, the increases in residence time indicated that once bound, the dead polymerase can remain stably associated with chromatin, even without PCNA. Consistent with our findings, evidence from *in vitro* polymerase assays demonstrated that DNA-bound polymerase-dead Pol δ apparently exchanges with unbound Pol δ at a lower rate than the WT Pol δ. This suggests that the dead polymerase might bind DNA more tightly than the WT enzyme (Hu et al., 2012). Thus, it appears that DNA polymerization may trigger dissociation of the Pol δ complex from chromatin, possibly by loosening the interaction with PCNA (“PCNA’s grip”) and/or with the DNA template. This would provide an important mechanism to aid the release of bound Pol δ from the genome to allow for the frequent recycling of Pol δ during lagging strand synthesis.

The essential role of the p125 catalytic subunit in the enzymatic function of Pol δ highlights the importance of maintaining adequate cellular p125 levels for proper genome replication. POLD1 gene transcription is regulated throughout the cell cycle, where relatively small increases in mRNA levels occur that peak in late G1/S phase, accompanied by corresponding modest increases in p125 protein levels (Zeng et al., 1994). The relatively long (10 h) half-life of p125 (Zeng et al., 1994) likely dampens the effects of cell-cycle-associated POLD1 transcriptional fluctuations on the cellular protein level. p125 Levels can also be transcriptionally regulated by WT p53 in response to DNA damage (Li and Lee, 2001). Intriguingly, our studies with ectopically expressed SNAP-Pol δ revealed that endogenous p125 levels adjusted according to the expression level of tagged p125 protein. Rather than having an additive effect on total cellular p125, ectopic expression of SNAP-tagged p125 led to a reduced expression of endogenous p125 (i.e., inversely proportional to SNAP-tagged p125 levels), regardless of whether catalytically active or inactive tagged p125 was expressed. Consequently, the total cellular p125 levels remained unchanged, suggesting that p125 is normally maintained at a relatively constant level and tightly regulated at the protein level. Significantly, the lack of an effect of WT SNAP-Pol δ replacement of endogenous p125 on cell growth rates and the inhibition of cell growth resulting from SNAP-Pol δ-Dead replacement indicate that the tagged p125 is likely incorporated into authentic functional Pol δ complexes (Figures 1E and 4C). Considering that the non-catalytic subunits could be in limited supply, fewer endogenous p125 molecules would be incorporated into Pol δ assemblies when tagged p125 is expressed, resulting in more uncomplexed, free endogenous p125. Thus, based on our findings, it is possible that p125 is protected from degradation when in complex with the other Pol δ subunits, whereas excess free p125 is degraded, providing a mechanism to prevent the formation of incomplete, inactive Pol δ assemblies. In support of this idea, it has been demonstrated in murine B-lymphocytes that depletion of the p68 subunit of Pol δ leads to reduced cellular p125 levels (Murga et al., 2016), indicating stoichiometric protection of p125 by p68.

We have used live-cell SMT to directly visualize human Pol δ holoenzyme assembly and disassembly, obtaining a detailed understanding of this process previously inaccessible by *in vitro* measurements. We have observed holoenzyme dynamics as they take place in the complex nuclear environment, via the interplay between the complete ensemble of replication factors present in living nuclei. Our analysis has revealed that although Pol δ holoenzyme assembly and disassembly in living cells proceed by an ordered predominant pathway, alternate pathways exist. The precise role of these alternate pathways in genomic replication awaits further investigation. It is expected that the predominant pathway we observe results in properly functioning Pol δ holoenzyme. Considering improper cellular Pol δ activity can result in genomic instability and cancer, our studies will be crucial to guide future studies to understand how altered Pol δ activity underlies these processes.

## STAR★METHODS

### LEAD CONTACT AND MATERIALS AVAILABILITY

Further information and requests for resources and reagents should be directed to and will be fulfilled by the Lead Contact, Carl Schildkraut (schildkr@aecom.yu.edu). Transfer of materials may require a material transfer agreement (MTA) to be signed.

### EXPERIMENTAL MODEL AND SUBJECT DETAILS

#### Cell Culture

LOX human (male) melanoma cells (Fodstad et al., 1988) were grown at 37°C and 5% CO_2_ in complete RPMI (high glucose RPMI supplemented with 10% FBS, 2 mM Glutamax (Fisher Scientific), 20mM HEPES, 100 I.U./mL Penicillin, and 100 μg/mL Streptomycin (Corning)). The line was authenticated by short tandem repeat (STR) profiling.

### METHOD DETAILS

#### Plasmid Construction

Doxycycline-inducible lentiviral plasmids used to express N-terminally Halo tagged PCNA, N-terminally SNAP tagged Pol δ (WT), and N-terminally SNAP tagged Pol δ Dead proteins were generated as follows. The inducible expression lentiviral vector pInducer10 (Meerbrey et al., 2011), a gift from W Guo, Albert Einstein College of Medicine, NY) was digested with AgeI and MluI and a linker containing NotI and XhoI sites inserted between the AgeI-MluI sites to generate pInducer10L. To create the Halo-PCNA expression construct, the Halo tag sequence was PCR amplified using the following primers: Forward 5′- AGTCGGTACCACCATGGCAGAAATCGGTACTGG –3′ and Reverse 5′- ACTGTGTACAGGCCGGAAATCTCAAGCGT –3′. The human PCNA cDNA sequence was excised from pEGFP-PCNA-IRES-puro2b (a gift from Daniel Gerlich; Addgene plasmid # 26461) (Held et al., 2010) and inserted along with the PCR amplified Halo tag sequence into the AgeI-NotI sites of pInducer10L to generate pInducer10-Halo-PCNA. The puromycin selective marker in pInducer10-Halo-PCNA was then replaced with a neomycin selective marker to generate pInd-Halo-PCNA. To create the SNAP-Pol δ (WT) expression construct, the SNAP tag sequence was PCR amplified using the following primers: Forward 5′- AGTCGGTACCACCATGGCAGAAATCGGTACTGG –3′ and Reverse 5′- ACTGTGTACAGGCCGGAAATCTCAAGCGT –3′. The human POLD1 cDNA sequence (Rosenbluh et al., 2016) was PCR amplified using the following primers: Forward 5′- AGTCGCGGCCGCCGGCCACATGGATGGCAAGCGGCGGCCA-3′ and Reverse 5′- AGTCCTCGAGTCACCAGGCCTCAGGTCCAGGGGGTC –3′, and inserted along with the PCR amplified SNAP tag sequence into the AgeI-NotI sites of pInducer10L to generate pInducer10-SNAP-Pol δ. The puromycin selective marker in pInducer10-SNAP-Pol δ was then replaced with a blasticidin selective marker to generate pInd-SNAP-Pol δ. To create the SNAP-Pol Dead expression construct, three alanine substitutions (D402A, D602A and D757A) at aspartates essential for Pol δ polymerase (Hu et al., 2012; Nicolas et al., 2016) and exonuclease activity (Meng et al., 2009; Schmitt et al., 2009) were introduced into the wild-type Pol δ sequence in pInd-SNAP-Pol δ using a geneblock (IDT), to generate pInd-SNAP-Pol δ Dead. All constructs were sequenced to confirm that unintended mutations were not introduced during PCR and cloning.

#### Generation of Halo-PCNA, SNAP-Pol δ, and SNAP-Pol δ Dead Stable Cell Lines

Stable LOX cell lines inducibly expressing Halo-PCNA and SNAP-Pol δ proteins were generated by lentiviral transduction. Cells were first infected with lentiviral particles containing pIND-Halo-PCNA and single cell clonal colonies were selected in 400μg/mL Geneticin (G418). To generate dual expression cell lines co-expressing Halo-PCNA and SNAP-Pol δ WT or SNAP-Pol δ Dead, we infected an individual clone of Halo-PCNA expressing LOX cells with lentiviral particles containing pIND-SNAP-Pol δ WT or pIND-SNAP-Pol δ Dead. Single cell clonal colonies of Halo-PCNA-expressing cells stably transduced with SNAP-Pol δ particles were then selected with 400μg/mL Geneticin and 4μg/mL blasticidin. Halo-PCNA and SNAP-Pol δ proteins were expressed in stable lines by induction with 1μg/ml Doxycycline.

#### Immunoblotting

Cells were harvested by trypsinization, suspended in complete RPMI, washed with PBS, then pelleted and flash frozen in liquid N_2_ and stored at −80°C. For SDS-PAGE, pellets were thawed on ice and lysed by resuspending in Laemmli Buffer (60 mM Tris-HCl pH 6.8, 400 mM 2-mercaptoethanol, 2% SDS, 10% glycerol, 0.01% bromophenol blue) to a final concentration of 10^6^ cells ml^−1^. Lysates were denatured (5min/100°C) and passed through a 25-gauge needle (5x) then spun for 2 min at full speed in a microfuge. Aliquots of lysate corresponding to 10^5^ cells were resolved on 4%–15% gradient SDS-PAGE gels (BioRad), proteins transferred to nitrocellulose membrane and blocked in PBS with 5% Blotting-grade Blocker (BioRad) and 0.1%Tween-20. Membranes were then incubated with primary antibodies diluted in PBS with 5% Blotting-grade Blocker. Primary antibodies used were: anti-human DNA Pol δ p125 (mouse monoclonal, Santa Cruz), anti-human PCNA (mouse monoclonal, Abcam), anti-SNAP tag (rabbit polyclonal, New England Biolabs), anti-Halo tag (mouse monoclonal, Promega), anti-α-Tubulin (mouse monoclonal, Sigma), and anti-actin (rabbit polyclonal, Sigma). Following incubation with primary antibodies membranes were washed with PBS + 0.1% Tween 20. Membranes were then incubated with fluorescently labeled Goat Anti-Mouse IRDye 680LT (Li-Cor) and Goat Anti-Rabbit IRDye 800CW (Li-Cor) secondary antibodies then washed in PBS + 0.1% Tween 20. Immunoblots were then imaged on an Odyssey Lc Infrared scanner (Li-Cor). Band intensities were quantified using ImageStudio software (Li-Cor).

#### Live-Cell Fluorescent Labeling of Halo-PCNA, SNAP-Pol δ, and SNAP-Pol δ Dead in LOX Cells

Two days prior to imaging, cells (2.3 ×10^5^ cells) stably expressing Halo-PCNA and SNAP-Pol δ, or SNAP-Pol δ Dead were plated in selective media on 35 mm MatTek imaging dishes. 24 hours prior to imaging 1 μg/ml Doxycycline was added to the cells and incubated at 37°C. Immediately prior to imaging, cells were incubated at 37°C with 0.4nM JF549-HTL (Janelia Labs) and 10nm SNAP-Cell 647-SiR (New England Biolabs) for a total of 15 and 60 minutes, respectively. Cells were then washed 3 times with 1x PBS, replaced with complete RPMI and further incubated for 30 minutes at 37°C to remove unincorporated dye. Cells were then washed 2 times with 1x PBS and placed in L-15 imaging media (Life technologies) + 10% FBS for imaging. For T2AA treatment, LOX cells co-expressing Halo-PCNA and SNAP-Pol δ were treated for 4 hours immediately prior to labeling with 20μM T2AA, labeled as above in the presence of 20mM T2AA, then two-color imaged in the presence of 20μM T2AA.

#### Live-Cell Single Molecule Imaging of Halo-PCNA, SNAP-Pol δ, and SNAP-Pol δ Dead in LOX Cells

All imaging sessions were carried out with asynchronous cultures at room temperature. Only cells displaying a punctate nuclear distribution pattern of PCNA, identifying them as S phase cells (Chagin et al., 2016), were imaged. Cells were continuously illuminated using 532nm (13 W/cm^2^, Coherent) and 640nm (9.5 W/cm^2^, Coherent) lasers for JF549-HTL and SNAP-Cell 647-SiR imaging respectively. Time-lapse two dimensional images of single molecules were acquired with a customized inverted Nikon Eclipse Ti microscope with a 150x oil-immersion objective lens (Nikon, 1.49 NA). Sequential dual color images of live cell nuclei were acquired for ~22 minutes using 500ms exposures on an EMCCD camera (iXon, Andor) with a 512 × 512 pixel field of view (final pixel size of 84nm) and a high speed filter wheel (~169ms dead time) that continuously alternated between the SNAP-Cell 647-SiR (labeled SNAP-Pol δ and SNAP-Pol δ Dead) and JF549-HTL (labeled Halo-PCNA) channels. The effective time resolution for each channel using such a system was 0.75Hz.

#### Image Processing and Single Molecule Tracking of Halo-PCNA, SNAP-Pol δ, and SNAP-Pol δ Dead in LOX Cells

Time-lapse movies of acquired images were de-interlaced to separate the two imaging channels and processed to subtract background in ImageJ using a rolling ball radius of 50 pixels. Background subtracted movies of separate imaging channels were subjected to Multi-Target Tracking (MTT) to resolve the trajectories of individual molecules (Sergé et al., 2008) using a Gui based implementation SLIMfast (Normanno et al., 2015). Localization of individual molecules was achieved by fitting the Point Spread function (PSF) from a discrete single spot with a 2D Gaussian function. Tracking of single molecule genomic binding events was performed by connecting of Halo-PCNA, SNAP-Pol δ or SNAP-Pol δ Dead localizations between consecutive frames. Tracking was performed using evalSPT (Normanno et al., 2015) based upon a maximum expected diffusion constant of 0.05 μm^2^/sec and allowing for 5 s gaps in trajectories due to blinking or missed localizations. A 2D projection map displaying the x,y positions of binding events for PCNA and Pol δ was generated using the average localizations of PSFs from individual binding trajectories of Halo-PCNA, SNAP-Pol δ, and SNAP-Pol δ Dead binding events over 22 minutes of imaging. Nuclear Halo-PCNA, SNAP-Pol δ, and SNAP-Pol δ Dead tracks were identified based on the boundaries from 2D projection maps of binding events. Nuclear boundaries were confirmed via 2D projections of the summed nuclear Halo-PCNA fluorescent signal after 22 minutes of imaging and confirmed using bright field imaging. Tracks that fell outside of the nucleus were excluded. Photobleach rates for single fluorescent dyes were then determined for each background-subtracted movie based upon the exponential decay of the global fluorescence signal of genomic bound Halo-PCNA, SNAP-Pol δ, and SNAP-Pol δ Dead. The average photobleach rates for JF549-HTL dye and the SNAP-Cell 647-SiR dye were 166.8 s and 99.3 s, respectively.

### QUANTIFICATION AND STATISTICAL ANALYSIS

#### Analysis of Colocalization and Temporal Order of Binding for Halo-PCNA and SNAP-Pol δ WT/Dead

Custom MATLAB scripts were used to identify SNAP-Pol δ WT/Dead genomic binding events lasting at > 1.34 s that temporally colocalized within 47nm of a Halo-PCNA molecule. The binding order was defined from co-localized Halo-PCNA and SNAP-Pol δ WT/ Dead tracks as follows. The “arrival” point was determined by the frame at which either SNAP-Pol δ WT/Dead or Halo-PCNA first appeared in a colocalized track. The “departure” point was the frame where signals disappeared. SNAP-Pol δ WT/Dead arrival and departure times (Figures 3D, 4E, and S1) were then set relative to the appearance and disappearance of Halo-PCNA. Order of binding was divided into three categories; SNAP-Pol δ WT/Dead arriving/departing before, at the same time or after Halo-PCNA.

#### Determination of PCNA-Halo and SNAP-Pol δ Chromatin Binding Residence Times

Genomic binding residence time was determined by plotting a survival curve (1-Cumulative Density Function, 1-CDF) of the track-lengths of nuclear bound Halo-PCNA, SNAP-Pol δ, and SNAP-Pol δ Dead in each cell. Single and double-exponential models were then fitted to these 1-CDF plots to determine the residence times. In general, fitting of the 1-CDF curves predominantly showed two populations of residences times consisting of both long-lived stable (> 1.7 s) and short-lived unstable (< 1.7 s) genomic binding events. A previous study examining chromatin binding of Sox2 found that unstable (residence time < 1 s) and stable (residence time > 1 s) binding arose from non-specific and specific interactions with the genome, respectively (Chen et al., 2014).

Global comparisons of both unstable (T_1_ population) and stable (T_2_ population) residence times, and proportions of molecules participating in unstable and stable residence events were conducted by taking the global residence time and the proportion of molecules participating in these residence events for each cell. A two-sample Kruskal-Wallis test was used to determine pairwise significance.

#### Analysis of SNAP-Pol δ Binding Hubs

2D projection maps of SNAP-Pol δ WT/Dead binding events were expanded 10-fold in the X and Y directions yielding a final pixel size of 8.4nm. Areas of high SNAP-Pol δ WT/Dead binding densities were determined by counting the number of binding events within an octagon window (diameter 168nm) as it was raster scanned across the expanded 2D projection map of the nucleus. Contiguous octagon windows centered on an individual pixel containing at least 3 SNAP-Pol δ WT/Dead binding events were defined and labeled as hubs. A two-sample Kruskal-Wallis test was used to determine pairwise significance.

The total number of hubs per cell were then normalized to the total number of SNAP-Pol δ WT/Dead binding events per cell. This normalized amount of hubs were then multiplied to produce the number of SNAP-Pol δ hubs formed per 1,000 binding events (i.e., Number of SNAP-Pol δ hubs) for each cell. Overall significance was determined with a two-sample Kruskal-Wallis test to determine pairwise significance.

To quantify binding latency times in hubs, tracks of binding events in individual hubs were further analyzed to determine the time periods between single SNAP-Pol δ binding events. 1-CDF survival plots of all SNAP-Pol δ latency times in hubs accumulated from 59 cells (Pol δ WT) and 13 cells (Pol δ Dead) were fit with a single, double, and triple exponential decay model. In general, fitting of the 1-CDF curves predominantly showed three populations of latency times consisting of short (~6–10 s), intermediate (~23–27 s) and long (~160–230 s) time periods. Overall significance was determined using a two-sided Student’s t test.

#### Statistical Analysis

Statistical details of experiments can be found in the figure legends. Statistical tests were performed using built-in scripts in MATLAB v 2014b (MathWorks, Natick, MA). Two-sample Kruskal-Wallis test and two-sided Student’s t test were used to determine pairwise comparisons.

### DATA AND CODE AVAILABILITY

Annotated Halo-PCNA and SNAP-PolD (WT/Dead) molecular localization, binding trajectory, residence time, and binding hub datasets generated during this study are available at MendeleyData. https://doi.org/10.17632/c62m7cm88v.1. The custom MATLAB-based SMT Analysis code generated during this study is available at https://github.com/Coleman-Laboratory/SMT-Data-and-code.

## Supplementary Material

Video S1

Video S2

Video S3

Video S4

Supplemental figures

## Figures and Tables

**KEY RESOURCES TABLE:** 

REAGENT or RESOURCE	SOURCE	IDENTIFIER
Antibodies		
Mouse monoclonal anti-human DNA Pol δ p125	Santa Cruz	Cat#sc-17776; RRID: AB_675487
Mouse monoclonal anti-human PCNA	Abcam	Cat#ab29; RRID: AB_303394
Rabbit polyclonal anti-SNAP tag	NEB	Cat#P9310; RRID: AB_10631145
Mouse monoclonal anti-Halo tag	Promega	Cat#G921A; RRID: AB_2688011
Rabbit polyclonal and anti-actin	Sigma	Cat#A2066; RRID: AB_476693
Goat polyclonal anti Rabbit IRDye800CW	Li-Cor	Cat#926-32211; RRID: AB_621843
Goat polyclonal anti-Mouse IRDye 680LT	Li-Cor	Cat#926-68020; RRID: AB_10706161
Mouse monoclonal anti-α-Tubulin	Sigma	Cat#T5168; RRID: AB_477579
Bacterial and Virus Strains		
*Escherichia coli* Stable competent cells	NEB	Cat#C3040H
Chemicals, Peptides, and Recombinant Proteins		
Doxycycline hyclate	Sigma	Cat#D9891
Puromycin	Sigma	Cat#P8833
Geneticin	Life Technologies	Cat#10131-035
Blasticidin	Millipore	Cat#203350
Leibovitz L-15 medium	Life Technologies	Cat#21083027
Q5 Hot Start High-Fidelity DNA Polymerase	NEB	Cat#M0493
JF549-HTL	Janelia Labs	JF549-HTL
SNAP-Cell 647-SiR	NEB	Cat#S9102S
T2 amino alcohol (T2AA)	Tocris	Cat#4723
XtremeGene HP transfection reagent	Roche	Cat#06 366 244 001
Critical Commercial Assays		
QIAquick PCR Purification Kit	QIAGEN	28104
QIAquick Gel Extraction Kit	QIAGEN	28704
Deposited Data		
Halo-PCNA and SNAP-PolD (WT/Dead) molecular localizations, binding trajectories, residence times, and binding hubs	This paper	MendeleyData. https://doi.org/10.17632/c62m7cm88v.1.
Experimental Models: Cell Lines		
LOX human melanoma cells	Laboratory of Lifeng Xu	RRID: CVCL_1381
Oligonucleotides		
Primer: Halo Forward: AGTCGGTACCACCATGGCAGAAATCGGTACTGG	This paper	N/A
Primer: Halo Reverse: ACTGTGTACAGGCCGGAAATCTCAAGCGT	This paper	N/A
Primer: POLD1 Forward: AGTCGCGGCCGCCGGCCACATGGATGGCAAGCGGCGGCCA	This paper	N/A
Primer: POLD1 Reverse: AGTCCTCGAGTCACCAGGCCTCAGGTCCAGGGGGTC	This paper	N/A
Primer: SNAP Forward: AGTCGGTACCACCATGGCAGAAATCGGTACTGG	This paper	N/A
Primer: SNAP Reverse: ACTGTGTACAGGCCGGAAATCTCAAGCGT	This paper	N/A
pInducer Linker Top: CCGGTTCCAGGTACATGGTGCGGCCGCTATCGATTGAATTCTCTCGAGTA	This paper	N/A
pInducer Linker Bottom: CGCGTACTCGAGAGAATTCAATCGATAGCGGCCGCACCATGTACCTGGAA	This paper	N/A
Primer: Neo Forward: AGTCGGCGCGCCTTAACGGATCCGAAATTCC	This paper	N/A
Primer: Neo Reverse: ACTGTTAATTAATCAGAAGAACTCGTCAAGAAGGCG	This paper	N/A
Primer: Blast Forward: ACTGCCACAACCATGGCCAAGCCTTTGTCTCAAG	This paper	N/A
Primer: Blast Reverse: ACTGTTAATTAATTAGCCCTCCCACACATAACCAG	This paper	N/A
Recombinant DNA		
pEGFP-PCNA-IRES-puro2b	Held et al., 2010	Addgene Plasmid # 26461; RRID: Addgene_26461
pInducer10	Meerbrey et al., 2011	Addgene Plasmid #44011; RRID: Addgene_44011
PolD1_pLX307 (PolD1 cDNA)	Rosenbluh et al., 2016	Addgene Plasmid #98358; RRID: Addgene_98358
pInducer10L	This paper	N/A
pInd-SNAP-Pol δ	This paper	N/A
pInd-Halo-PCNA	This paper	N/A
Software and Algorithms		
ImageStudio	Li-Cor	https://www.licor.com/bio/image-studio/
ImageJ	NIH	https://imagej.nih.gov
SLIMfast	Normanno et al., 2015	https://github.com/Coleman-Laboratory/SMT-Data-and-code
evalSPT	Normanno et al., 2015	https://github.com/Coleman-Laboratory/SMT-Data-and-code
MATLAB	MathWorks	https://www.mathworks.com/products/matlab.html
MATLAB based SMT Analysis code	This paper	https://github.com/Coleman-Laboratory/SMT-Data-and-code

## Highlights

- Human Pol δ interaction with the genome is highly dynamic
- *In vivo* Pol δ holoenzyme assembly/disassembly follows an ordered predominant pathway
- Alternate holoenzyme pathways likely reflect challenged genome copying
- Pol δ p125 is maintained at a constant cellular level, controlling Pol δ levels *in vivo*
