# Supplementary material for: Dynamic Assembly and Disassembly of the Human DNA Polymerase δ Holoenzyme on the Genome In Vivo
Source: Cell Rep. Author manuscript; Available in PMC 2020 Oct 30. (PMC7597369; doi:10.1016/j.celrep.2019.12.101)
Supplement: Supplemental figures [file NIHMS1637637-supplement-Supplemental_figures.pdf]

Cell Reports, Volume *30*

Supplemental Information

Dynamic Assembly and Disassembly of the Human DNA Polymerase δ Holoenzyme on the Genome *In Vivo*

William C. Drosopoulos, David A. Vierra, Charles A. Kenworthy, Robert A. Coleman, and Carl L. Schildkraut

Figure S1

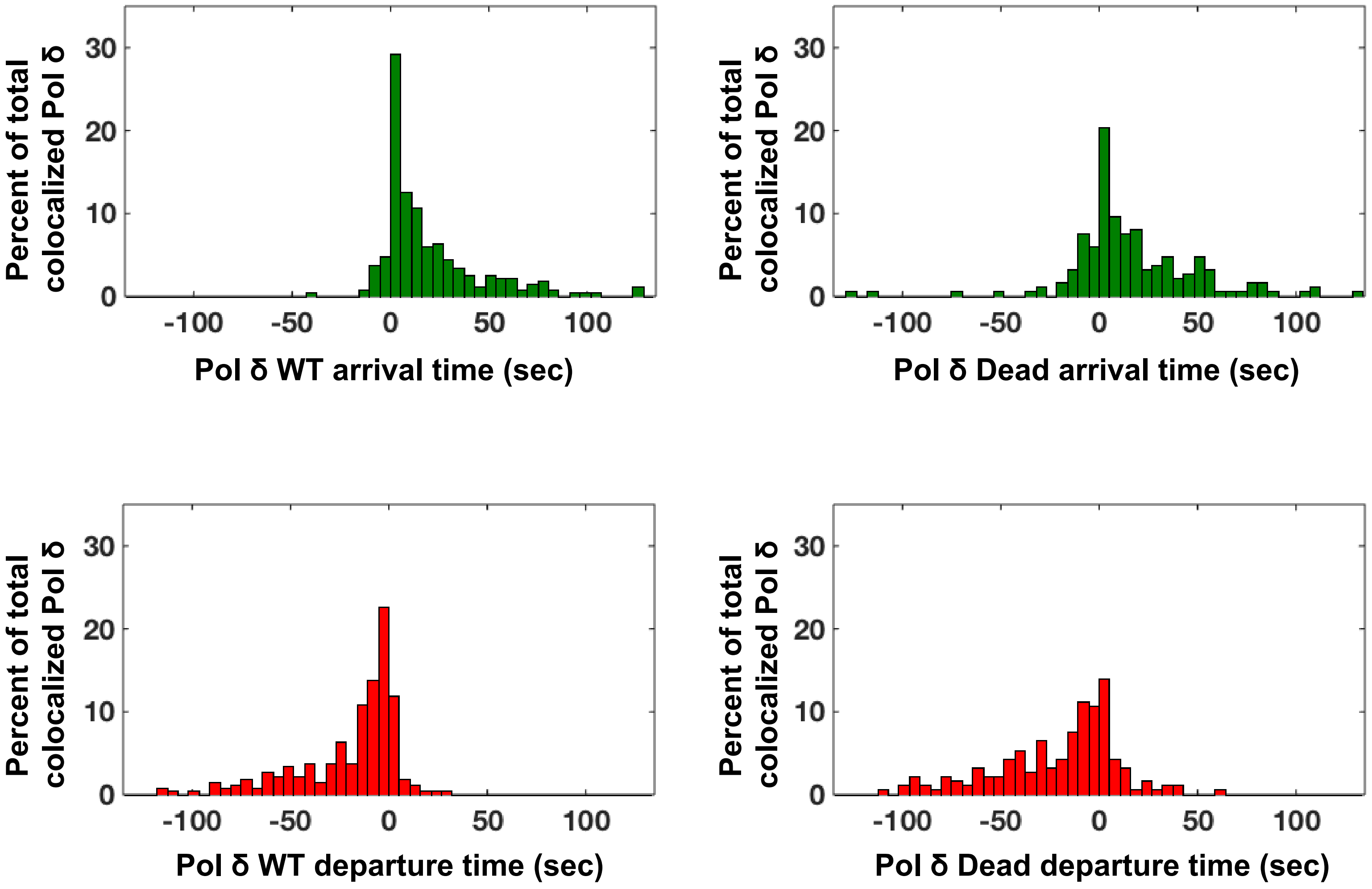

Figure S1: Genomic loading and unloading of SNAP-Pol δ WT and SNAP-Pol δ Dead colocalized with Halo-PCNA related to Figures 3 and 4. Histograms showing the percent of total SNAP-Pol δ WT (left) or SNAP-Pol δ Dead (right) molecules loaded (arrival, top) and unloaded (departure, bottom) before, at the same time, or after, co-expressed Halo-PCNA molecules during observed genomic colocalization events shown in Figures 3C and 4D. Times are relative to PCNA arrival (top panels) or departure (bottom panels).

Figure S2

	Arrival (sec)	Departure (sec)	Overlap (sec)
**Pol δ WT**			
**Arrive before PCNA**			
Depart before PCNA	-3.35 (-1.94 – -4.75)	-6.03 (-3.34 – -8.62)	2.68 (1.03 – 4.33)
Depart after PCNA	-5.36 (-1.68 – -9.04)	1.34 (-4.79 – 7.65)	3.35 (1.24 – 5.45)
**Arrive after PCNA**			
Depart before PCNA	17.42 (13.69 – 21.15)	-20.1 (-15.8 – -24.4)	5.36 (4.74 – 5.98)
Depart after PCNA	4.02 (2.03 – 6.0)	3.35 (0.87 – 5.83)	5.36 (1.89 – 8.83)
**Pol δ Dead**			
**Arrive before PCNA**			
Depart before PCNA	-8.04 (-5.35 – -10.73)	-18.76 (-5.3 – -32.2)	7.37 (5.13 – 9.6)
Depart after PCNA	-12.06 (-8.11 – -16.01)	6.7 (-3.12 – 16.52)	9.38 (4.15 – 14.61)
**Arrive after PCNA**			
Depart before PCNA	19.43 (13.5 – 25.36)	-28.14 (-22.01 – -34.27)	6.7 (4.86 – 8.54)
Depart after PCNA	9.38 (2.96 – 15.8)	5.36 (2.31 – 8.41)	6.7 (3.97 – 9.44)

Figure S2: Median time intervals of arrival, colocalization and departure of Pol δ/ PCNA during colocalization events related to Figures 3 and 4.
Table summarizing colocalization events depicted in Figures 3D and 4E. Median time intervals of arrival, colocalization and departure of Pol δ/ PCNA during colocalization events are shown with 95% confidence intervals

Figure S3

Tagged protein	Co-expressed tagged protein	Residence Time Short binders (T_1) (sec)	Percent in Population	Residence Time Long binders (T_2) (sec)	Percent in Population
Halo-PCNA	SNAP-Pol δ WT p125	1.92 ± 0.1	87.3 ± 2.8	20.0 ± 9	12.7 ± 2.8
SNAP-Pol δ WT p125	Halo-PCNA	1.76 ± 0.09	96.2 ± 1.9	12.4 ± 4	3.8 ± 1.9
Halo-PCNA	SNAP-Pol δ Dead p125	2.00 ± 0.03	84 ± 1.2	43.9 ± 7.5	16 ± 1.2
SNAP-Pol δ Dead p125	Halo-PCNA	2.11 ± 0.07	84 ± 2.9	22.4 ± 7.4	16 ± 2.9

Figure S3: Genome residence times of SNAP-Pol δ WT, SNAP-Pol δ Dead and Halo-PCNA related to Figure 5. Genomic binding residence time was determined by plotting a survival curve (1-CDF) of the track-lengths of nuclear bound Halo-PCNA, WT SNAP-Pol δ, and SNAP-Pol δ Dead in each cell. Single and double-exponential models were then fitted to these 1-CDF plots to determine the median residence times (T). The fitted curves identified two predominant populations of residences times consisting of both short (<1.7 sec), unstable binding events (T1 population) and long (>1.7 sec), stable binding events (T2 population). Binding events from 59 nuclei of SNAP-Pol δ WT (C11) cells from 4 independent experiments and 13 nuclei of SNAP-Pol δ Dead (C2) cells from 2 independent experiments were analyzed. Median T1 and T2 values ± SD are shown. Percent in population = the median percent ± SD of the total binding events (tracks) represented by each population.

Figure S4

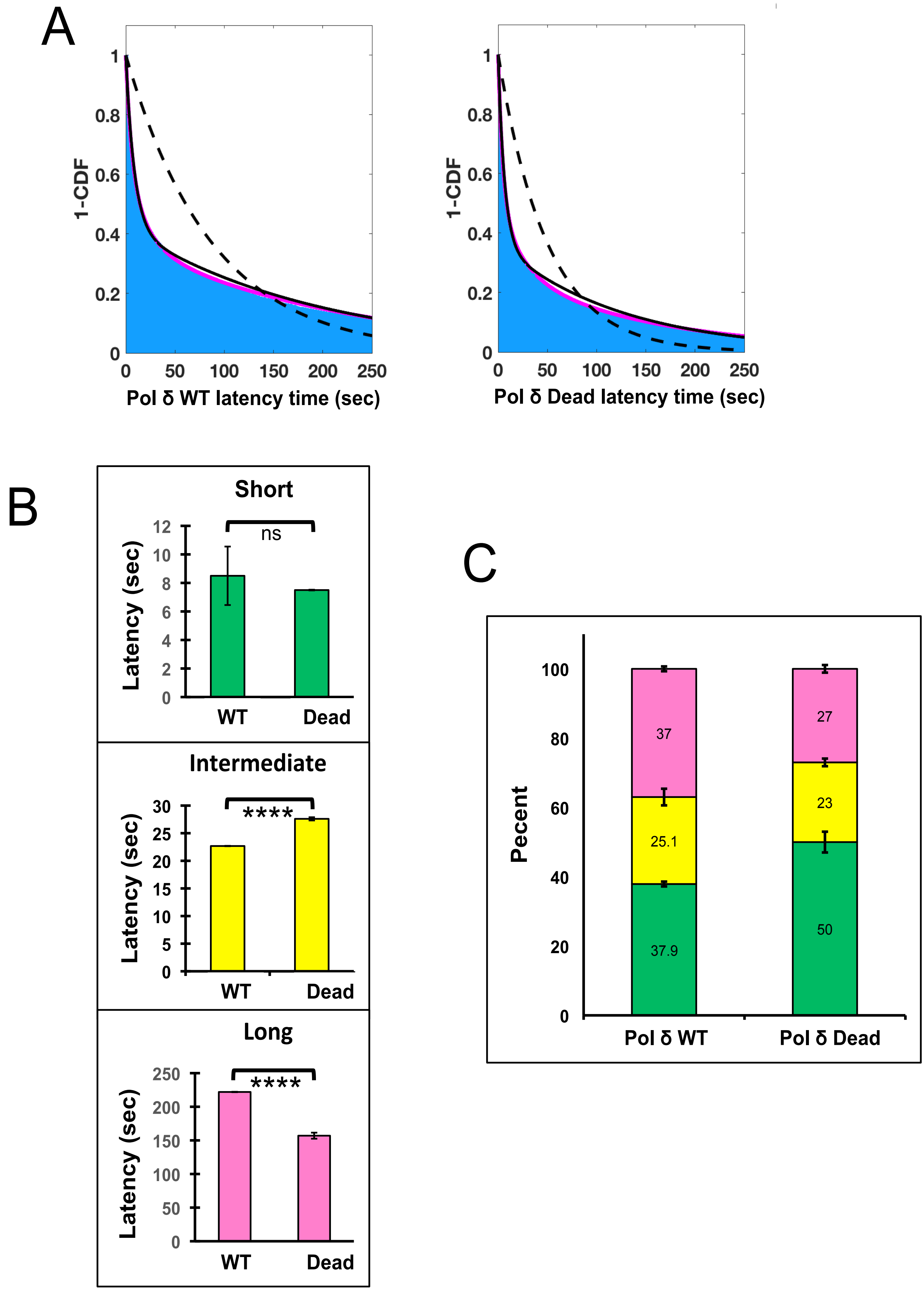
A
1
0.8
0.6
0.4
0.2
0
1-CDF
0
50
100
150
200
250
Pol δ WT latency time (sec)
Pol δ Dead latency time (sec)
B
Short
Latency (sec)
12
10
8
6
4
2
0
ns
WT
Dead
Intermediate
30
25
20
15
10
5
0
****
WT
Dead
Long
250
200
150
100
50
0
****
WT
Dead
C
Pecent
100
80
60
40
20
0
37
25.1
37.9
27
23
50
Pol δ WT
Pol δ Dead

Figure S4: Latency times of Pol δ binding events in hubs related to Figure 6.

(A) Binding latency times in hubs were determined from 1−Cumulative Distribution Function (1−CDF) plots of nuclear SNAP-Pol δ WT and Dead bound to chromatin fitted to a single (black dashed), two-component (black solid) or three-component (magenta solid) exponential decay model. (B) The 1−CDF curves identified three predominant populations of latency times: one of short (~6-10 seconds), one of intermediate (~23-27 seconds) and one of long (~160-230 seconds) time periods. Asterisks (****) indicate a p-value of <0.0001. (C) Histogram indicating the percent of total binding events in the short (green), intermediate (yellow) and long (pink) latency populations. Binding events from 59 nuclei of SNAP-Pol δ WT (C11) cells from 4 independent experiments and 13 nuclei of SNAP-Pol δ Dead (C2) cells from 2 independent experiments were analyzed. A two-sided Student's t-test was used to determine significance.
